# Author Correction: Relationships of stomatal morphology to the environment across plant communities

**DOI:** 10.1038/s41467-024-44730-4

**Published:** 2024-01-10

**Authors:** Congcong Liu, Lawren Sack, Ying Li, Jiahui Zhang, Kailiang Yu, Qiongyu Zhang, Nianpeng He, Guirui Yu

**Affiliations:** 1 Key Laboratory of Ecology and Environment in Minority Areas (Minzu University of China), National Ethnic Affairs Commission, 100081 Beijing, China; 2 College of Life and Environmental Sciences, Minzu University of China, 100081 Beijing, China; 3 Key Laboratory of Ecosystem Network Observation and Modeling, Institute of Geographic Sciences and Natural Resources Research, Chinese Academy of Sciences, 100101 Beijing, China; 4 Department of Ecology and Evolutionary Biology, University of California, Los Angeles, CA 90025 USA; 5 Department of Ecology and Evolutionary Biology, Princeton University, Princeton, NJ 08540 USA; 6 Center for Ecological Research, Northeast Forestry University, 150040 Harbin, China; 7 Earth Critical Zone and Flux Research Station of Xing’an Mountains, Chinese Academy of Sciences, 165200 Daxing’anling, China; 8 College of Resources and Environment, University of Chinese Academy of Sciences, 100049 Beijing, China

**Keywords:** Biogeography, Biodiversity, Plant ecology, Macroecology

Correction to: *Nature Communications* 10.1038/s41467-023-42136-2, published online 19 October 2023

The original version of this Article contained an error in Ref. 66, which was incorrectly given as: ‘Urgoiti Otazua, J. et al. No complementarity no gain—net diversity effects on tree productivity occur once complementarity emerges during early stand development. *Ecol. Lett.* **25**, 851–862 (2022)’. The correct Ref. 66 is: ‘Lai, J., Zou, Y., Zhang, S., Zhang, X. & Mao, L. glmm.hp: an R package for computing individual effect of predictors in generalized linear mixed models. *J. Plant Ecol*. **15**, 1302–1307 (2022).’. This has been corrected in the PDF and HTML versions of the Article.

