# Supplementary material for: Relationships of stomatal morphology to the environment across plant communities
Source: Nat Commun. 2023 Oct 19;14:6629. doi: 10.1038/s41467-023-42136-2 (PMC10587080; doi:10.1038/s41467-023-42136-2)
Supplement: Supplementary file 1 — Supplementary Information [file 41467_2023_42136_MOESM1_ESM.pdf]

Supplementary Information

Relationships of stomatal morphology to environment across plant communities

Liu et al.

Table S1 Stomatal traits and community-weighted trait moments of this study.

	Unit	Description and significance	Diagram
**Stomatal traits**			
Stomatal density (SD)	pores mm^{-2}	The number of stomata per leaf area. There is typically a trade-off between density and size., Generally, SD is positively correlated with maximum photosynthetic capacity and water use per leaf area, and negatively correlated with water retention and water use-efficiency.	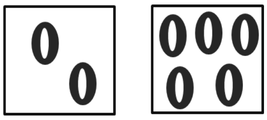
Stomatal length (SL)	μm	Equivalent to guard cell length, and an index of stomatal size. Small stomata respond more quickly to changing environments.	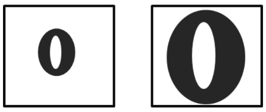
Stomatal pore index (SPI)	%	The fraction of leaf surface covered by stomata. The SPI, like SD, confers higher maximum photosynthetic capacity and water use per leaf area, and negatively correlated with water retention and water use-efficiency	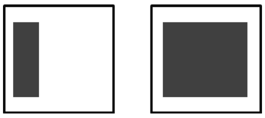
**Stomatal trait moments**			
Community-weighted mean (CWM)	/	Functional identity or dominance, the optimal trait value at local abiotic conditions, and it was related to the "mass ratio hypothesis".	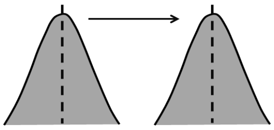
Community-weighted variance (CWV)	/	Functional diversity, especially the dispersion in the distribution of traits. It was related to the "niche complementarity hypothesis"	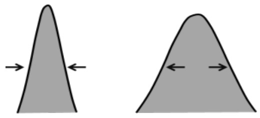
Community-weighted skewness (CWS)	/	Functional rarity, depicting the asymmetric nature of the trait distribution, and it was related to the "niche complementarity hypothesis"	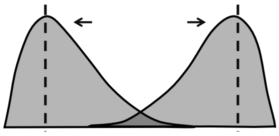
Community-weighted kurtosis (CWK)	/	Functional evenness, lower CWK value represented higher functional evenness, and it was related to the "niche complementarity hypothesis"	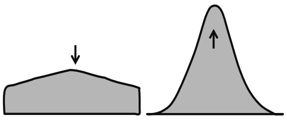

Table S2 Summary of environmental variables used in this study.

Full name	Abbr.	Unit	Note
Aridity index (MAP/PET)	AI	Unitless	
Annual Mean Temperature	bio1	°C	
Mean Diurnal Range	bio2	°C	Mean of monthly (max temp - min temp)
Isothermality	bio3	Unitless	(BIO2/BIO7) (×100)
Temperature Seasonality	bio4	Unitless	standard deviation ×100
Max Temperature of Warmest Month	bio5	°C	
Min Temperature of Coldest Month	bio6	°C	
Temperature Annual Range	bio7	°C	BIO5-BIO6
Mean Temperature of Wettest Quarter	bio8	°C	
Mean Temperature of Driest Quarter	bio9	°C	
Mean Temperature of Warmest Quarter	bio10	°C	
Mean Temperature of Coldest Quarter	bio11	°C	
Annual Precipitation	bio12	mm	
Precipitation of Wettest Month	bio13	mm	
Precipitation of Driest Month	bio14	mm	
Precipitation Seasonality	bio15	Unitless	Coefficient of Variation
Precipitation of Wettest Quarter	bio16	mm	
Precipitation of Driest Quarter	bio17	mm	
Precipitation of Warmest Quarter	bio18	mm	
Precipitation of Coldest Quarter	bio19	mm	
Growing-season temperature	T_{gs}	°C	
Growing-season precipitation	P_{gs}	mm	
Growing-season aridity index	AI_{gs}	Unitless	
Total Nitrogen	TN	% of weight	
pH	pH	Unitless	
Sand content	SAND	% of weight	
Silt content	SILT	% of weight	
Clay content	CLAY	% of weight	
Soil Moisture	SM	m^3/m^3	
Bulk density	BD	cg/cm^3	

Table S3 Summary of the effects of vegetation type on the stomatal trait moments (two-tailed statistical tests). Statistical analysis was performed using linear mixed effects models with vegetation type as a fixed factor, and plot nested within sites as random factors. Source data are provided as a Source Data file.

	Variable	DF	T value	P value
SD_mean	Vegetation type	53.48	-5.56	< 0.001
SL_mean	Vegetation type	54.937	1.594	0.117
SPI_mean	Vegetation type	54.234	-3.78	< 0.001
SD_var	Vegetation type	56.484	-6.904	< 0.001
SL_var	Vegetation type	56.417	-0.839	0.405
SPI_var	Vegetation type	56.022	-5.914	< 0.001
SD_skew	Vegetation type	57.589	-1.026	0.309
SL_skew	Vegetation type	54.833	-2.347	0.02255
SPI_skew	Vegetation type	57.214	-0.741	0.462
SD_kurt	Vegetation type	64.880	1.099	0.276
SL_kurt	Vegetation type	54.578	-2.117	0.0388
SPI_kurt	Vegetation type	64.493	0.930	0.356

Table S4 Standardized effect sizes (SESs) for the variance in CWMs and for the mean in CWVs, CWSs, and CWKs. SD_mean, community-weighted mean of stomatal density; SL_mean, community-weighted mean of stomatal length; SPI_mean, community-weighted mean of stomatal pore index. SD_var, community-weighted variance of stomatal density; SL_var, community-weighted variance of stomatal length; SPI_var, community-weighted variance of stomatal pore index. SD_skew, community-weighted skewness of stomatal density; SL_skew, community-weighted skewness of stomatal length; SPI_ skew, community-weighted skewness of stomatal pore index. SD_kurt, community-weighted kurtosis of stomatal density; SL_kurt, community-weighted kurtosis of stomatal length; SPI_kurt, community-weighted kurtosis of stomatal pore index. Source data are provided as a Source Data file.

	Trait moments	Expected	All	Forests	Grasslands
SES_{var}	SD_mean	+	2.62	6.78	-3.21
SES_{var}	SL_mean	+	3.93	4.98	2.97
SES_{var}	SPI_mean	+	3.31	6.61	-0.77
SES_{mean}	SD_var	-	-1.50	17.31	-8.93
SES_{mean}	SL_var	-	-0.30	2.76	-1.83
SES_{mean}	SPI_var	-	-0.03	16.07	-7.21
SES_{mean}	SD_skew	-	-1.22	0.07	-1.76
SES_{mean}	SL_skew	-	-3.50	-0.37	-5.03
SES_{mean}	SPI_skew	-	-1.62	-0.65	-2.21
SES_{mean}	SD_kurt	-	-1.77	-2.18	-1.62
SES_{mean}	SL_kurt	-	-2.05	-2.29	-1.95
SES_{mean}	SPI_kurt	-	-1.83	-1.61	-2.04

Table S5 Relationships between community-weighted mean of stomatal density (SD_CWM) and environmental variables. All linear regressions are estimated using the linear mixed model with plot nested within sites as a random factor. Slope, the slope of the regression between stomatal trait moments and environments. LL, the lower limit of the 95% confidence interval for slope. UL, the upper limit of the 95% confidence interval for slope. R^2m, marginal R^2 (fixed effects only); R^2c, conditional R^2 (both fixed and random effects). DF, degree of freedom. Different vegetation types and environmental variables were filled with different background colors. See Table S2 for environmental variable abbreviations. Source data are provided as a Source Data file.

	All								Forests								Grasslands							
Y	Slope	LL	UL	DF	T value	P value	R^2c	R^2m	Slope	LL	UL	DF	T value	P value	R^2c	R^2m	Slope	LL	UL	DF	T value	P value	R^2c	R^2m
AI	0.27	0.15	0.38	55.04	4.55	0.00	0.19	0.78	0.07	-0.15	0.28	26.07	0.60	0.56	0.01	0.79	0.26	-0.03	0.55	27.00	1.74	0.09	0.06	0.61
bio1	0.02	0.01	0.02	55.70	5.18	0.00	0.23	0.78	0.01	0.00	0.02	26.03	2.60	0.02	0.16	0.79	0.01	0.00	0.02	27.00	2.07	0.05	0.09	0.61
bio2	-0.05	-0.07	-0.03	56.11	-5.28	0.00	0.23	0.77	-0.03	-0.06	-0.01	26.06	-2.47	0.02	0.15	0.79	-0.05	-0.09	0.00	27.00	-2.03	0.05	0.08	0.61
bio3	0.03	-0.87	0.94	55.09	0.07	0.95	0.00	0.79	0.51	-0.59	1.61	26.06	0.90	0.38	0.02	0.79	-0.11	-1.10	0.89	27.00	-0.21	0.84	0.00	0.61
bio4	-0.02	-0.04	-0.01	55.67	-2.82	0.01	0.09	0.79	-0.02	-0.04	-0.01	26.01	-2.48	0.02	0.15	0.79	-0.01	-0.03	0.01	27.00	-0.62	0.54	0.01	0.61
bio5	0.02	0.01	0.03	53.61	2.96	0.00	0.13	0.79	0.02	-0.01	0.05	26.07	1.51	0.14	0.06	0.79	0.00	0.00	0.01	27.00	0.97	0.34	0.02	0.61
bio6	0.01	0.01	0.01	56.28	4.77	0.00	0.19	0.77	0.01	0.00	0.01	26.03	2.52	0.02	0.16	0.79	0.01	0.00	0.02	27.00	2.37	0.03	0.11	0.61
bio7	-0.01	-0.01	0.00	56.08	-3.59	0.00	0.13	0.78	-0.01	-0.01	0.00	26.02	-2.55	0.02	0.16	0.79	0.00	-0.01	0.00	27.00	-0.99	0.33	0.02	0.61
bio8	0.02	0.01	0.03	54.10	4.22	0.00	0.21	0.79	0.03	0.01	0.05	26.08	3.01	0.01	0.21	0.79	0.01	0.00	0.02	27.00	1.22	0.23	0.03	0.61
bio9	0.01	0.01	0.02	56.28	4.68	0.00	0.18	0.78	0.01	0.00	0.01	26.03	2.57	0.02	0.16	0.79	0.01	0.00	0.02	27.00	2.27	0.03	0.10	0.61
bio10	0.02	0.01	0.03	54.15	4.04	0.00	0.19	0.79	0.02	0.00	0.04	26.05	2.34	0.03	0.14	0.79	0.01	0.00	0.02	27.00	1.22	0.23	0.03	0.61
bio11	0.01	0.01	0.02	56.28	4.68	0.00	0.18	0.78	0.01	0.00	0.01	26.03	2.57	0.02	0.16	0.79	0.01	0.00	0.02	27.00	2.27	0.03	0.10	0.61
bio12	0.00	0.00	0.00	55.46	5.00	0.00	0.21	0.77	0.00	0.00	0.00	26.10	1.29	0.21	0.05	0.79	0.00	0.00	0.00	27.00	2.31	0.03	0.10	0.61
bio13	0.00	0.00	0.00	55.02	5.00	0.00	0.22	0.78	0.00	0.00	0.00	26.04	1.29	0.21	0.05	0.79	0.00	0.00	0.00	27.00	1.91	0.07	0.07	0.61
bio14	0.01	0.00	0.01	56.86	2.78	0.01	0.07	0.78	0.00	0.00	0.01	26.37	0.59	0.56	0.01	0.79	0.03	0.00	0.07	27.00	1.80	0.08	0.07	0.61
bio15	-0.63	-0.90	-0.37	55.13	-4.76	0.00	0.21	0.78	-0.32	-0.76	0.12	26.23	-1.41	0.17	0.05	0.79	-0.45	-0.83	-0.07	27.00	-2.33	0.03	0.10	0.61
bio16	0.00	0.00	0.00	54.88	5.33	0.00	0.24	0.78	0.00	0.00	0.00	25.99	1.48	0.15	0.06	0.79	0.00	0.00	0.00	27.00	1.84	0.08	0.07	0.61
bio17	0.00	0.00	0.00	56.65	3.09	0.00	0.08	0.78	0.00	0.00	0.00	26.28	0.73	0.47	0.02	0.79	0.01	0.00	0.02	27.00	2.20	0.04	0.09	0.61

bio18	0.00	0.00	0.00	54.85	5.40	0.00	0.25	0.78	0.00	0.00	0.00	25.97	1.55	0.13	0.07	0.79	0.00	0.00	0.00	27.00	1.84	0.08	0.07	0.61
bio19	0.00	0.00	0.00	56.65	3.09	0.00	0.08	0.78	0.00	0.00	0.00	26.28	0.73	0.47	0.02	0.79	0.01	0.00	0.02	27.00	2.20	0.04	0.09	0.61
T_{gs}	0.03	0.01	0.04	54.46	3.84	0.00	0.17	0.79	0.03	0.00	0.05	26.19	1.97	0.06	0.10	0.79	0.01	-0.01	0.02	27.00	1.16	0.26	0.03	0.61
P_{gs}	0.00	0.00	0.00	55.59	4.98	0.00	0.20	0.77	0.00	0.00	0.00	26.11	1.32	0.20	0.05	0.79	0.00	0.00	0.00	27.00	2.48	0.02	0.12	0.61
AI_{gs}	0.23	0.11	0.35	54.82	3.86	0.00	0.16	0.78	0.05	-0.18	0.28	26.07	0.43	0.67	0.01	0.79	0.09	-0.10	0.28	27.00	0.88	0.39	0.02	0.61
TN	-0.04	-0.27	0.20	55.51	-0.32	0.75	0.00	0.79	-0.50	-0.71	-0.28	25.79	-4.53	0.00	0.36	0.79	0.08	-0.41	0.56	27.00	0.31	0.76	0.00	0.61
pH	-0.10	-0.15	-0.06	54.59	-4.25	0.00	0.18	0.78	0.02	-0.13	0.16	26.02	0.21	0.83	0.00	0.79	0.00	-0.12	0.12	27.00	0.01	1.00	0.00	0.61
SAND	-0.01	-0.01	-0.01	54.54	-4.46	0.00	0.20	0.79	-0.01	-0.01	0.00	26.10	-1.28	0.21	0.05	0.79	0.00	-0.01	0.00	27.00	-1.41	0.17	0.04	0.61
SILT	0.01	0.01	0.02	54.21	3.53	0.00	0.15	0.79	0.01	0.00	0.02	26.21	1.62	0.12	0.07	0.79	0.00	0.00	0.01	27.00	1.38	0.18	0.04	0.61
CLAY	0.02	0.01	0.02	55.27	3.41	0.00	0.12	0.78	0.00	-0.01	0.02	26.10	0.28	0.78	0.00	0.79	0.00	-0.01	0.02	27.00	0.51	0.62	0.01	0.61
SM	0.10	-0.73	0.92	57.76	0.23	0.82	0.00	0.79	-0.16	-1.01	0.68	26.79	-0.38	0.71	0.00	0.79	-2.28	-4.38	-0.18	27.00	-2.13	0.04	0.09	0.61
BD	0.00	-0.01	0.00	54.47	-1.66	0.10	0.04	0.79	0.01	0.00	0.02	25.91	2.43	0.02	0.15	0.79	0.00	-0.01	0.00	27.00	-0.63	0.54	0.01	0.61

Table S6 Relationships between community-weighted mean of stomatal length (SL_CWM) and environmental variables. All linear regressions are estimated using the linear mixed model with plot nested within sites as a random factor. Slope, the slope of the regression between stomatal trait moments and environments. LL, the lower limit of the 95% confidence interval for slope. UL, the upper limit of the 95% confidence interval for slope. R^2m, marginal R^2 (fixed effects only); R^2c, conditional R^2 (both fixed and random effects). DF, degree of freedom. Different vegetation types and environmental variables were filled with different background colors. See Table S2 for environmental variable abbreviations. Source data are provided as a Source Data file.

	All								Forests								Grasslands							
Y	Slope	LL	UL	DF	T value	P value	R^2c	R^2m	Slope	LL	UL	DF	T value	P value	R^2c	R^2m	Slope	LL	UL	DF	T value	P value	R^2c	R^2m
AI	-0.06	-0.10	-0.01	56.04	-2.57	0.01	0.07	0.76	-0.08	-0.16	-0.01	26.08	-2.11	0.04	0.12	0.85	0.02	-0.12	0.17	27.00	0.29	0.77	0.00	0.71
bio1	0.00	-0.01	0.00	57.24	-3.66	0.00	0.13	0.76	-0.01	-0.01	0.00	26.21	-5.55	0.00	0.46	0.84	0.00	0.00	0.01	27.00	1.20	0.24	0.04	0.71
bio2	0.01	0.00	0.02	57.23	3.18	0.00	0.10	0.76	0.02	0.01	0.03	26.17	3.84	0.00	0.31	0.84	-0.02	-0.04	0.00	27.00	-1.60	0.12	0.06	0.71
bio3	-0.62	-0.88	-0.35	55.14	-4.55	0.00	0.20	0.76	-0.55	-0.91	-0.19	26.06	-2.99	0.01	0.22	0.84	-0.84	-1.19	-0.48	27.00	-4.62	0.00	0.32	0.71
bio4	0.02	0.01	0.02	57.64	8.00	0.00	0.39	0.75	0.02	0.01	0.02	26.20	6.49	0.00	0.53	0.84	0.02	0.01	0.03	27.00	4.50	0.00	0.31	0.71
bio5	0.00	0.00	0.01	53.60	0.51	0.61	0.00	0.77	-0.01	-0.02	0.00	26.11	-2.52	0.02	0.16	0.84	0.01	0.00	0.01	27.00	3.12	0.00	0.19	0.71
bio6	0.00	-0.01	0.00	58.66	-5.50	0.00	0.23	0.75	0.00	-0.01	0.00	26.21	-5.91	0.00	0.49	0.84	0.00	-0.01	0.00	27.00	-0.94	0.36	0.02	0.71
bio7	0.01	0.00	0.01	58.43	7.54	0.00	0.35	0.74	0.00	0.00	0.01	26.21	6.20	0.00	0.51	0.84	0.01	0.00	0.01	27.00	3.99	0.00	0.26	0.71
bio8	0.00	0.00	0.00	54.18	-0.33	0.74	0.00	0.77	-0.01	-0.02	-0.01	26.05	-3.40	0.00	0.26	0.84	0.01	0.00	0.01	27.00	2.95	0.01	0.17	0.71
bio9	0.00	-0.01	0.00	58.78	-5.87	0.00	0.25	0.75	0.00	-0.01	0.00	26.21	-6.08	0.00	0.50	0.84	0.00	-0.01	0.00	27.00	-1.44	0.16	0.05	0.71
bio10	0.00	0.00	0.00	54.39	-0.48	0.63	0.00	0.77	-0.01	-0.02	-0.01	26.15	-3.58	0.00	0.28	0.84	0.01	0.00	0.01	27.00	2.95	0.01	0.17	0.71
bio11	0.00	-0.01	0.00	58.78	-5.87	0.00	0.25	0.75	0.00	-0.01	0.00	26.21	-6.08	0.00	0.50	0.84	0.00	-0.01	0.00	27.00	-1.44	0.16	0.05	0.71
bio12	0.00	0.00	0.00	56.79	-3.20	0.00	0.10	0.76	0.00	0.00	0.00	26.16	-3.17	0.00	0.23	0.84	0.00	0.00	0.00	27.00	0.69	0.49	0.01	0.71
bio13	0.00	0.00	0.00	56.23	-2.87	0.01	0.09	0.76	0.00	0.00	0.00	26.07	-3.02	0.01	0.22	0.84	0.00	0.00	0.00	27.00	0.81	0.43	0.02	0.71
bio14	0.00	0.00	0.00	57.81	-2.60	0.01	0.06	0.76	0.00	0.00	0.00	26.29	-1.91	0.07	0.10	0.84	0.00	-0.02	0.02	27.00	0.19	0.85	0.00	0.71
bio15	0.12	0.02	0.22	56.20	2.33	0.02	0.06	0.76	0.22	0.08	0.37	26.23	2.95	0.01	0.20	0.84	-0.11	-0.30	0.08	27.00	-1.10	0.28	0.03	0.71
bio16	0.00	0.00	0.00	55.96	-2.66	0.01	0.08	0.76	0.00	0.00	0.00	26.05	-2.99	0.01	0.22	0.84	0.00	0.00	0.00	27.00	1.03	0.31	0.03	0.71

bio17	0.00	0.00	0.00	57.71	-2.92	0.00	0.07	0.76	0.00	0.00	0.00	26.24	-2.25	0.03	0.13	0.84	0.00	0.00	0.00	27.00	0.13	0.90	0.00	0.71
bio18	0.00	0.00	0.00	55.90	-2.64	0.01	0.08	0.76	0.00	0.00	0.00	26.02	-2.98	0.01	0.22	0.85	0.00	0.00	0.00	27.00	1.03	0.31	0.03	0.71
bio19	0.00	0.00	0.00	57.71	-2.92	0.00	0.07	0.76	0.00	0.00	0.00	26.24	-2.25	0.03	0.13	0.84	0.00	0.00	0.00	27.00	0.13	0.90	0.00	0.71
T_{gs}	0.00	-0.01	0.00	54.72	-0.41	0.68	0.00	0.77	-0.01	-0.02	0.00	26.24	-2.90	0.01	0.20	0.84	0.01	0.00	0.02	27.00	3.19	0.00	0.19	0.71
P_{gs}	0.00	0.00	0.00	57.00	-3.33	0.00	0.10	0.76	0.00	0.00	0.00	26.17	-3.40	0.00	0.26	0.84	0.00	0.00	0.00	27.00	0.97	0.34	0.02	0.71
AI_{gs}	-0.06	-0.10	-0.01	55.48	-2.54	0.01	0.07	0.76	-0.07	-0.15	0.01	26.07	-1.68	0.11	0.08	0.85	-0.03	-0.12	0.06	27.00	-0.66	0.52	0.01	0.71
TN	0.11	0.04	0.19	56.15	2.93	0.00	0.09	0.76	0.19	0.10	0.27	26.00	4.50	0.00	0.38	0.84	0.32	0.12	0.52	27.00	3.14	0.00	0.19	0.71
pH	0.02	0.00	0.04	55.21	1.84	0.07	0.04	0.76	0.03	-0.02	0.09	26.03	1.26	0.22	0.05	0.85	-0.02	-0.07	0.04	27.00	-0.65	0.52	0.01	0.71
SAND	0.00	0.00	0.00	55.00	2.10	0.04	0.06	0.77	0.00	0.00	0.01	26.06	1.28	0.21	0.05	0.85	0.00	0.00	0.00	27.00	0.45	0.66	0.01	0.71
SILT	0.00	0.00	0.00	54.37	-1.41	0.16	0.03	0.77	0.00	-0.01	0.00	26.12	-0.16	0.88	0.00	0.85	0.00	-0.01	0.00	27.00	-0.94	0.36	0.02	0.71
CLAY	0.00	-0.01	0.00	56.00	-2.09	0.04	0.05	0.76	-0.01	-0.01	0.00	26.10	-1.93	0.06	0.10	0.85	0.00	0.00	0.01	27.00	0.94	0.35	0.02	0.71
SM	-0.02	-0.30	0.27	58.40	-0.11	0.91	0.00	0.77	-0.04	-0.35	0.28	26.57	-0.24	0.81	0.00	0.85	1.17	0.18	2.15	27.00	2.32	0.03	0.12	0.71
BD	0.00	0.00	0.00	54.82	0.13	0.90	0.00	0.77	0.00	-0.01	0.00	25.96	-3.41	0.00	0.27	0.85	0.00	0.00	0.01	27.00	1.72	0.10	0.07	0.71

Table S7 Relationships between community-weighted mean of stomatal pore index (SPI_CWM) and environmental variables. All linear regressions are estimated using the linear mixed model with plot nested within sites as a random factor. Slope, the slope of the regression between stomatal trait moments and environments. LL, the lower limit of the 95% confidence interval for slope. UL, the upper limit of the 95% confidence interval for slope. R^2m, marginal R^2 (fixed effects only); R^2c, conditional R^2 (both fixed and random effects). DF, degree of freedom. Different vegetation types and environmental variables were filled with different background colors. See Table S2 for environmental variable abbreviations. Source data are provided as a Source Data file.

	All								Forests								Grasslands							
Y	Slope	LL	UL	DF	T value	P value	R^2c	R^2m	Slope	LL	UL	DF	T value	P value	R^2c	R^2m	Slope	LL	UL	DF	T value	P value	R^2c	R^2m
AI	0.15	0.04	0.26	55.60	2.60	0.01	0.07	0.80	-0.05	-0.23	0.14	26.02	-0.50	0.62	0.01	0.79	0.26	-0.10	0.62	27.00	1.40	0.17	0.05	0.76
bio1	0.01	0.00	0.02	55.68	2.85	0.01	0.09	0.80	0.00	-0.01	0.01	26.03	0.21	0.84	0.00	0.79	0.02	0.01	0.03	27.00	2.98	0.01	0.19	0.76
bio2	-0.03	-0.05	-0.01	56.06	-3.38	0.00	0.11	0.79	-0.01	-0.03	0.02	26.01	-0.75	0.46	0.02	0.79	-0.09	-0.14	-0.04	27.00	-3.63	0.00	0.25	0.76
bio3	-1.15	-1.89	-0.40	55.25	-3.02	0.00	0.11	0.80	-0.44	-1.37	0.49	26.00	-0.92	0.37	0.03	0.79	-1.94	-2.90	-0.97	27.00	-3.93	0.00	0.28	0.76
bio4	0.01	-0.01	0.02	55.75	0.77	0.44	0.01	0.80	0.00	-0.02	0.02	26.00	0.10	0.92	0.00	0.79	0.03	0.01	0.05	27.00	2.58	0.02	0.15	0.76
bio5	0.02	0.01	0.03	53.35	4.25	0.00	0.23	0.81	0.00	-0.02	0.03	26.02	0.24	0.82	0.00	0.79	0.02	0.01	0.03	27.00	3.76	0.00	0.26	0.76
bio6	0.00	0.00	0.01	56.33	1.64	0.11	0.03	0.80	0.00	0.00	0.00	26.02	0.08	0.93	0.00	0.79	0.01	-0.01	0.02	27.00	1.11	0.28	0.03	0.76
bio7	0.00	-0.01	0.00	56.12	-0.05	0.96	0.00	0.80	0.00	-0.01	0.01	26.01	-0.05	0.96	0.00	0.79	0.01	0.00	0.02	27.00	1.92	0.07	0.09	0.76
bio8	0.02	0.01	0.03	53.77	5.04	0.00	0.27	0.80	0.01	-0.01	0.03	25.98	1.33	0.19	0.05	0.79	0.02	0.01	0.03	27.00	3.94	0.00	0.28	0.76
bio9	0.00	0.00	0.01	56.33	1.40	0.17	0.02	0.80	0.00	-0.01	0.01	26.02	0.07	0.95	0.00	0.79	0.00	-0.01	0.02	27.00	0.60	0.55	0.01	0.76
bio10	0.02	0.01	0.03	53.78	4.56	0.00	0.24	0.80	0.01	-0.01	0.02	26.01	0.59	0.56	0.01	0.79	0.02	0.01	0.03	27.00	3.94	0.00	0.28	0.76
bio11	0.00	0.00	0.01	56.33	1.40	0.17	0.02	0.80	0.00	-0.01	0.01	26.02	0.07	0.95	0.00	0.79	0.00	-0.01	0.02	27.00	0.60	0.55	0.01	0.76
bio12	0.00	0.00	0.00	55.89	2.63	0.01	0.07	0.80	0.00	0.00	0.00	26.08	-0.32	0.75	0.00	0.79	0.00	0.00	0.00	27.00	2.31	0.03	0.12	0.76
bio13	0.00	0.00	0.00	55.51	2.71	0.01	0.08	0.80	0.00	0.00	0.00	26.03	-0.39	0.70	0.00	0.79	0.00	0.00	0.00	27.00	2.05	0.05	0.10	0.76
bio14	0.00	0.00	0.01	57.09	1.25	0.22	0.01	0.80	0.00	0.00	0.00	26.31	-0.45	0.66	0.01	0.79	0.04	-0.01	0.09	27.00	1.73	0.10	0.07	0.76
bio15	-0.43	-0.68	-0.18	55.42	-3.35	0.00	0.12	0.80	-0.01	-0.39	0.38	26.17	-0.04	0.97	0.00	0.79	-0.69	-1.12	-0.26	27.00	-3.12	0.00	0.20	0.76
bio16	0.00	0.00	0.00	55.34	3.13	0.00	0.11	0.80	0.00	0.00	0.00	25.96	-0.12	0.91	0.00	0.79	0.00	0.00	0.00	27.00	2.18	0.04	0.11	0.76
bio17	0.00	0.00	0.00	56.89	1.31	0.19	0.02	0.80	0.00	0.00	0.00	26.24	-0.52	0.60	0.01	0.79	0.01	0.00	0.02	27.00	1.93	0.06	0.09	0.76
bio18	0.00	0.00	0.00	55.31	3.20	0.00	0.11	0.80	0.00	0.00	0.00	25.93	-0.03	0.98	0.00	0.79	0.00	0.00	0.00	27.00	2.18	0.04	0.11	0.76

bio19	0.00	0.00	0.00	56.89	1.31	0.19	0.02	0.80	0.00	0.00	0.00	26.24	-0.52	0.60	0.01	0.79	0.01	0.00	0.02	27.00	1.93	0.06	0.09	0.76
T_{gs}	0.03	0.02	0.04	54.04	4.44	0.00	0.22	0.80	0.01	-0.02	0.03	26.13	0.50	0.62	0.01	0.79	0.03	0.02	0.05	27.00	4.19	0.00	0.30	0.76
P_{gs}	0.00	0.00	0.00	55.98	2.55	0.01	0.07	0.80	0.00	0.00	0.00	26.09	-0.39	0.70	0.00	0.79	0.00	0.00	0.00	27.00	2.79	0.01	0.17	0.76
AI_{gs}	0.11	0.00	0.23	55.40	1.95	0.06	0.05	0.80	-0.04	-0.23	0.15	26.01	-0.41	0.69	0.01	0.79	-0.02	-0.25	0.22	27.00	-0.13	0.90	0.00	0.77
TN	0.12	-0.09	0.32	55.70	1.13	0.26	0.01	0.80	-0.21	-0.43	0.02	25.84	-1.77	0.09	0.09	0.79	0.71	0.19	1.24	27.00	2.64	0.01	0.15	0.76
pH	-0.06	-0.11	-0.02	55.13	-2.76	0.01	0.09	0.80	0.06	-0.06	0.18	25.96	1.01	0.32	0.03	0.79	-0.03	-0.18	0.11	27.00	-0.45	0.65	0.01	0.77
SAND	-0.01	-0.01	0.00	55.15	-2.94	0.00	0.10	0.80	0.00	-0.01	0.00	26.03	-0.68	0.50	0.01	0.79	0.00	-0.01	0.00	27.00	-0.86	0.40	0.02	0.76
SILT	0.01	0.00	0.02	54.91	2.64	0.01	0.09	0.80	0.01	0.00	0.02	26.18	1.79	0.09	0.09	0.79	0.00	-0.01	0.01	27.00	0.53	0.60	0.01	0.77
CLAY	0.01	0.00	0.02	55.63	2.02	0.05	0.05	0.80	0.00	-0.02	0.01	26.04	-0.79	0.44	0.02	0.79	0.01	-0.01	0.03	27.00	1.02	0.32	0.03	0.76
SM	0.04	-0.69	0.77	57.97	0.10	0.92	0.00	0.80	-0.21	-0.92	0.50	26.72	-0.57	0.58	0.01	0.79	-0.47	-3.22	2.27	27.00	-0.34	0.74	0.00	0.77
BD	0.00	-0.01	0.00	55.09	-1.29	0.20	0.02	0.80	0.00	-0.01	0.01	25.88	0.51	0.62	0.01	0.79	0.00	0.00	0.01	27.00	0.84	0.41	0.02	0.76

Table S8 Relationships between community-weighted variance of stomatal density (SD_CWV) and environmental variables. All linear regressions are estimated using the linear mixed model with plot nested within sites as a random factor. Slope, the slope of the regression between stomatal trait moments and environments. LL, the lower limit of the 95% confidence interval for slope. UL, the upper limit of the 95% confidence interval for slope. R^2m, marginal R^2 (fixed effects only); R^2c, conditional R^2 (both fixed and random effects). DF, degree of freedom. Different vegetation types and environmental variables were filled with different background colors. See Table S2 for environmental variable abbreviations. Source data are provided as a Source Data file.

	All								Forests								Grasslands							
Y	Slope	LL	UL	DF	T value	P value	R^2c	R^2m	Slope	LL	UL	DF	T value	P value	R^2c	R^2m	Slope	LL	UL	DF	T value	P value	R^2c	R^2m
AI	0.77	0.47	1.07	56.17	5.06	0.00	0.22	0.77	-0.08	-0.46	0.30	26.14	-0.39	0.70	0.00	0.73	1.15	0.22	2.08	27.00	2.43	0.02	0.12	0.67
bio1	0.04	0.02	0.06	56.79	3.81	0.00	0.14	0.77	0.02	0.00	0.03	26.15	2.04	0.05	0.10	0.73	0.01	-0.03	0.05	27.00	0.54	0.59	0.01	0.67
bio2	-0.11	-0.17	-0.05	57.07	-3.74	0.00	0.13	0.77	-0.04	-0.09	0.02	26.18	-1.36	0.18	0.05	0.73	-0.04	-0.20	0.12	27.00	-0.52	0.61	0.01	0.67
bio3	0.23	-2.19	2.65	55.76	0.19	0.85	0.00	0.78	1.10	-0.81	3.00	26.08	1.13	0.27	0.04	0.73	0.88	-2.42	4.18	27.00	0.52	0.61	0.01	0.67
bio4	-0.05	-0.09	0.00	56.30	-2.01	0.05	0.05	0.78	-0.03	-0.07	0.00	26.10	-1.92	0.07	0.09	0.73	-0.02	-0.09	0.05	27.00	-0.61	0.55	0.01	0.67
bio5	0.04	0.01	0.06	54.46	2.29	0.03	0.08	0.78	0.03	-0.02	0.08	26.16	1.26	0.22	0.04	0.73	0.00	-0.03	0.03	27.00	-0.14	0.89	0.00	0.67
bio6	0.02	0.01	0.03	57.22	3.41	0.00	0.11	0.77	0.01	0.00	0.02	26.14	1.89	0.07	0.09	0.73	0.01	-0.02	0.05	27.00	0.79	0.44	0.01	0.67
bio7	-0.02	-0.03	0.00	56.76	-2.62	0.01	0.07	0.78	-0.01	-0.02	0.00	26.12	-1.88	0.07	0.09	0.73	-0.01	-0.04	0.02	27.00	-0.79	0.44	0.02	0.67
bio8	0.04	0.02	0.07	54.94	3.10	0.00	0.13	0.78	0.04	0.00	0.08	26.11	2.14	0.04	0.11	0.73	0.00	-0.03	0.03	27.00	0.11	0.92	0.00	0.67
bio9	0.02	0.01	0.04	57.19	3.39	0.00	0.11	0.77	0.01	0.00	0.02	26.13	2.00	0.06	0.10	0.73	0.02	-0.02	0.05	27.00	0.89	0.38	0.02	0.67
bio10	0.04	0.02	0.07	55.12	3.16	0.00	0.13	0.78	0.03	0.00	0.07	26.16	1.88	0.07	0.09	0.73	0.00	-0.03	0.03	27.00	0.11	0.92	0.00	0.67
bio11	0.02	0.01	0.04	57.19	3.39	0.00	0.11	0.77	0.01	0.00	0.02	26.13	2.00	0.06	0.10	0.73	0.02	-0.02	0.05	27.00	0.89	0.38	0.02	0.67
bio12	0.00	0.00	0.00	56.73	5.14	0.00	0.22	0.77	0.00	0.00	0.00	26.22	0.56	0.58	0.01	0.73	0.00	0.00	0.00	27.00	2.63	0.01	0.14	0.67
bio13	0.00	0.00	0.01	56.15	5.58	0.00	0.25	0.77	0.00	0.00	0.00	26.14	0.60	0.55	0.01	0.73	0.01	0.00	0.01	27.00	2.93	0.01	0.16	0.67
bio14	0.01	0.00	0.03	57.91	2.82	0.01	0.07	0.77	0.00	-0.01	0.01	26.55	0.31	0.76	0.00	0.73	0.09	-0.04	0.22	27.00	1.39	0.18	0.04	0.67
bio15	-1.41	-2.15	-0.66	56.50	-3.69	0.00	0.14	0.78	-0.31	-1.10	0.49	26.40	-0.75	0.46	0.02	0.73	-0.25	-1.63	1.12	27.00	-0.36	0.72	0.00	0.67
bio16	0.00	0.00	0.00	56.03	6.01	0.00	0.28	0.77	0.00	0.00	0.00	26.07	0.73	0.47	0.02	0.73	0.00	0.00	0.00	27.00	2.83	0.01	0.15	0.67
bio17	0.00	0.00	0.01	57.74	3.12	0.00	0.08	0.77	0.00	0.00	0.00	26.45	0.46	0.65	0.01	0.73	0.02	0.00	0.05	27.00	1.73	0.09	0.07	0.67
bio18	0.00	0.00	0.00	55.99	6.05	0.00	0.29	0.77	0.00	0.00	0.00	26.04	0.76	0.45	0.02	0.73	0.00	0.00	0.00	27.00	2.83	0.01	0.15	0.67

bio19	0.00	0.00	0.01	57.74	3.12	0.00	0.08	0.77	0.00	0.00	0.00	26.45	0.46	0.65	0.01	0.73	0.02	0.00	0.05	27.00	1.73	0.09	0.07	0.67
T_{gs}	0.06	0.02	0.10	55.41	2.86	0.01	0.11	0.78	0.04	-0.01	0.09	26.29	1.67	0.11	0.07	0.73	-0.01	-0.06	0.05	27.00	-0.19	0.85	0.00	0.67
P_{gs}	0.00	0.00	0.00	56.88	5.05	0.00	0.21	0.77	0.00	0.00	0.00	26.24	0.61	0.55	0.01	0.73	0.00	0.00	0.00	27.00	2.73	0.01	0.15	0.67
AI_{gs}	0.72	0.42	1.03	55.69	4.68	0.00	0.21	0.77	-0.12	-0.52	0.27	26.13	-0.61	0.55	0.01	0.73	0.62	0.02	1.22	27.00	2.01	0.05	0.09	0.67
TN	0.42	-0.19	1.04	56.01	1.34	0.18	0.02	0.78	-0.78	-1.18	-0.38	25.92	-3.80	0.00	0.28	0.73	0.65	-0.95	2.26	27.00	0.79	0.43	0.02	0.67
pH	-0.33	-0.45	-0.21	55.86	-5.45	0.00	0.26	0.77	0.12	-0.13	0.37	26.07	0.93	0.36	0.02	0.73	-0.12	-0.51	0.27	27.00	-0.59	0.56	0.01	0.67
SAND	-0.03	-0.04	-0.01	55.74	-4.00	0.00	0.17	0.78	-0.01	-0.02	0.01	26.19	-0.83	0.41	0.02	0.73	0.00	-0.02	0.02	27.00	-0.26	0.80	0.00	0.67
SILT	0.03	0.01	0.05	55.13	2.78	0.01	0.10	0.78	0.02	0.00	0.04	26.32	1.55	0.13	0.06	0.73	0.00	-0.02	0.03	27.00	0.13	0.89	0.00	0.67
CLAY	0.04	0.02	0.07	56.30	3.66	0.00	0.13	0.78	0.00	-0.03	0.02	26.17	-0.32	0.75	0.00	0.73	0.01	-0.04	0.07	27.00	0.38	0.71	0.00	0.67
SM	0.64	-1.57	2.85	58.57	0.57	0.57	0.00	0.78	-0.68	-2.14	0.78	27.16	-0.91	0.37	0.02	0.73	0.71	-6.83	8.26	27.00	0.18	0.85	0.00	0.67
BD	-0.02	-0.03	-0.01	55.13	-3.02	0.00	0.11	0.78	0.02	0.00	0.03	25.97	2.35	0.03	0.14	0.73	-0.01	-0.03	0.01	27.00	-1.04	0.31	0.03	0.67

Table S9 Relationships between community-weighted variance of stomatal length (SL_CWV) and environmental variables. All linear regressions are estimated using the linear mixed model with plot nested within sites as a random factor. Slope, the slope of the regression between stomatal trait moments and environments. LL, the lower limit of the 95% confidence interval for slope. UL, the upper limit of the 95% confidence interval for slope. R^2m, marginal R^2 (fixed effects only); R^2c, conditional R^2 (both fixed and random effects). DF, degree of freedom. Different vegetation types and environmental variables were filled with different background colors. See Table S2 for environmental variable abbreviations. Source data are provided as a Source Data file.

	All								Forests								Grasslands							
Y	Slope	LL	UL	DF	T value	P value	R^2c	R^2m	Slope	LL	UL	DF	T value	P value	R^2c	R^2m	Slope	LL	UL	DF	T value	P value	R^2c	R^2m
AI	0.02	-0.20	0.24	57.71	0.17	0.86	0.00	0.63	-0.10	-0.43	0.24	26.20	-0.56	0.58	0.01	0.66	-0.19	-1.02	0.63	27.00	-0.46	0.65	0.01	0.65
bio1	-0.01	-0.02	0.01	58.31	-0.93	0.36	0.01	0.63	-0.02	-0.03	0.00	26.29	-2.50	0.02	0.14	0.65	0.01	-0.03	0.04	27.00	0.44	0.66	0.00	0.65
bio2	0.01	-0.03	0.05	58.85	0.53	0.60	0.00	0.63	0.04	0.00	0.08	26.27	1.81	0.08	0.08	0.65	-0.04	-0.17	0.08	27.00	-0.67	0.51	0.01	0.65
bio3	-2.45	-3.80	-1.11	57.27	-3.57	0.00	0.12	0.62	-1.76	-3.32	-0.20	26.19	-2.20	0.04	0.11	0.65	-3.44	-5.80	-1.09	27.00	-2.86	0.01	0.15	0.65
bio4	0.05	0.03	0.08	58.57	4.23	0.00	0.15	0.62	0.05	0.02	0.07	26.21	3.43	0.00	0.22	0.65	0.08	0.03	0.13	27.00	3.32	0.00	0.19	0.65
bio5	0.02	0.00	0.04	53.66	1.79	0.08	0.04	0.63	-0.02	-0.07	0.02	26.25	-0.88	0.39	0.02	0.66	0.02	0.00	0.05	27.00	1.94	0.06	0.08	0.65
bio6	-0.01	-0.02	0.00	59.78	-2.15	0.04	0.04	0.62	-0.01	-0.02	0.00	26.26	-2.94	0.01	0.18	0.65	-0.02	-0.04	0.01	27.00	-1.17	0.25	0.03	0.65
bio7	0.02	0.01	0.02	59.36	3.53	0.00	0.10	0.62	0.01	0.01	0.02	26.23	3.23	0.00	0.20	0.65	0.03	0.01	0.05	27.00	2.98	0.01	0.16	0.65
bio8	0.01	-0.01	0.03	54.40	1.30	0.20	0.02	0.63	-0.02	-0.06	0.01	26.18	-1.43	0.16	0.05	0.65	0.02	0.00	0.05	27.00	1.83	0.08	0.07	0.65
bio9	-0.01	-0.02	0.00	59.78	-2.39	0.02	0.05	0.62	-0.01	-0.02	0.00	26.27	-2.96	0.01	0.18	0.65	-0.02	-0.05	0.00	27.00	-1.62	0.12	0.06	0.65
bio10	0.01	-0.01	0.03	54.72	1.31	0.20	0.02	0.63	-0.02	-0.05	0.01	26.28	-1.33	0.20	0.04	0.65	0.02	0.00	0.05	27.00	1.83	0.08	0.07	0.65
bio11	-0.01	-0.02	0.00	59.78	-2.39	0.02	0.05	0.62	-0.01	-0.02	0.00	26.27	-2.96	0.01	0.18	0.65	-0.02	-0.05	0.00	27.00	-1.62	0.12	0.06	0.65
bio12	0.00	0.00	0.00	58.44	0.02	0.98	0.00	0.63	0.00	0.00	0.00	26.31	-1.02	0.32	0.03	0.65	0.00	0.00	0.00	27.00	0.18	0.86	0.00	0.65
bio13	0.00	0.00	0.00	57.66	0.35	0.73	0.00	0.63	0.00	0.00	0.00	26.20	-0.81	0.43	0.02	0.66	0.00	0.00	0.01	27.00	0.61	0.55	0.01	0.65
bio14	0.00	-0.01	0.01	60.91	0.04	0.97	0.00	0.63	0.00	-0.01	0.01	26.74	-0.53	0.60	0.01	0.65	0.04	-0.07	0.14	27.00	0.73	0.47	0.01	0.65
bio15	0.02	-0.49	0.53	57.64	0.09	0.93	0.00	0.63	0.36	-0.32	1.05	26.52	1.03	0.31	0.03	0.65	-0.03	-1.15	1.08	27.00	-0.06	0.96	0.00	0.65
bio16	0.00	0.00	0.00	57.27	0.47	0.64	0.00	0.63	0.00	0.00	0.00	26.11	-0.69	0.50	0.01	0.66	0.00	0.00	0.00	27.00	0.57	0.57	0.01	0.65
bio17	0.00	0.00	0.00	60.50	0.04	0.97	0.00	0.63	0.00	0.00	0.00	26.60	-0.52	0.61	0.01	0.66	0.00	-0.02	0.03	27.00	0.27	0.79	0.00	0.65

bio18	0.00	0.00	0.00	57.18	0.47	0.64	0.00	0.63	0.00	0.00	0.00	26.07	-0.69	0.49	0.01	0.66	0.00	0.00	0.00	27.00	0.57	0.57	0.01	0.65
bio19	0.00	0.00	0.00	60.50	0.04	0.97	0.00	0.63	0.00	0.00	0.00	26.60	-0.52	0.61	0.01	0.66	0.00	-0.02	0.03	27.00	0.27	0.79	0.00	0.65
T_{gs}	0.01	-0.01	0.04	55.42	0.97	0.33	0.01	0.63	-0.03	-0.07	0.01	26.53	-1.39	0.18	0.05	0.65	0.03	-0.01	0.07	27.00	1.62	0.12	0.06	0.65
P_{gs}	0.00	0.00	0.00	58.66	-0.03	0.98	0.00	0.63	0.00	0.00	0.00	26.33	-1.23	0.23	0.04	0.65	0.00	0.00	0.00	27.00	0.58	0.57	0.01	0.65
AI_{gs}	-0.04	-0.26	0.18	56.92	-0.33	0.75	0.00	0.63	-0.08	-0.42	0.26	26.18	-0.46	0.65	0.01	0.66	-0.34	-0.85	0.16	27.00	-1.32	0.20	0.04	0.65
TN	0.32	-0.05	0.70	58.18	1.68	0.10	0.03	0.62	0.29	-0.13	0.71	26.02	1.36	0.19	0.05	0.65	0.54	-0.76	1.83	27.00	0.81	0.43	0.02	0.65
pH	-0.02	-0.11	0.07	56.58	-0.36	0.72	0.00	0.63	0.03	-0.19	0.26	26.09	0.30	0.77	0.00	0.66	0.13	-0.19	0.45	27.00	0.81	0.42	0.02	0.65
SAND	0.00	-0.01	0.01	56.29	-0.35	0.72	0.00	0.63	0.00	-0.02	0.01	26.22	-0.68	0.50	0.01	0.66	0.01	-0.01	0.02	27.00	0.80	0.43	0.01	0.65
SILT	0.00	-0.02	0.01	55.27	-0.42	0.67	0.00	0.63	0.01	-0.01	0.03	26.37	0.92	0.36	0.02	0.65	-0.02	-0.03	0.00	27.00	-1.57	0.13	0.05	0.65
CLAY	0.01	-0.01	0.03	57.64	1.21	0.23	0.01	0.63	0.00	-0.02	0.02	26.25	0.08	0.94	0.00	0.66	0.03	-0.01	0.07	27.00	1.41	0.17	0.04	0.65
SM	0.04	-1.33	1.42	62.86	0.06	0.95	0.00	0.63	-0.28	-1.56	1.01	27.58	-0.42	0.68	0.00	0.65	2.63	-3.40	8.67	27.00	0.85	0.40	0.02	0.65
BD	0.00	-0.01	0.01	56.10	0.23	0.82	0.00	0.63	-0.01	-0.02	0.01	25.97	-0.91	0.37	0.02	0.66	0.02	0.00	0.03	27.00	2.20	0.04	0.10	0.65

Table S10 Relationships between community-weighted variance of stomatal pore index (SPI_CWV) and environmental variables. All linear regressions are estimated using the linear mixed model with plot nested within sites as a random factor. Slope, the slope of the regression between stomatal trait moments and environments. LL, the lower limit of the 95% confidence interval for slope. UL, the upper limit of the 95% confidence interval for slope. R^2m, marginal R^2 (fixed effects only); R^2c, conditional R^2 (both fixed and random effects). DF, degree of freedom. Different vegetation types and environmental variables were filled with different background colors. See Table S2 for environmental variable abbreviations. Source data are provided as a Source Data file.

	All								Forests								Grasslands							
Y	Slope	LL	UL	DF	T value	P value	R^2c	R^2m	Slope	LL	UL	DF	T value	P value	R^2c	R^2m	Slope	LL	UL	DF	T value	P value	R^2c	R^2m
AI	0.49	0.24	0.74	56.56	3.86	0.00	0.13	0.65	-0.07	-0.44	0.30	26.10	-0.39	0.70	0.00	0.71	0.23	-0.52	0.98	27.00	0.60	0.56	0.01	0.50
bio1	0.03	0.01	0.05	57.04	3.87	0.00	0.13	0.65	0.01	-0.01	0.03	26.10	1.37	0.18	0.05	0.70	0.02	-0.01	0.05	27.00	1.55	0.13	0.04	0.50
bio2	-0.10	-0.14	-0.05	57.76	-4.28	0.00	0.14	0.65	-0.03	-0.08	0.01	26.13	-1.36	0.18	0.05	0.70	-0.12	-0.23	-0.01	27.00	-2.17	0.04	0.08	0.50
bio3	-1.74	-3.55	0.08	55.59	-1.87	0.07	0.04	0.66	-0.05	-1.94	1.85	26.08	-0.05	0.96	0.00	0.71	-3.09	-5.25	-0.94	27.00	-2.81	0.01	0.12	0.50
bio4	-0.01	-0.04	0.03	56.54	-0.37	0.71	0.00	0.66	-0.02	-0.05	0.02	26.07	-1.00	0.33	0.03	0.70	0.06	0.01	0.11	27.00	2.31	0.03	0.09	0.50
bio5	0.04	0.02	0.07	52.85	4.12	0.00	0.19	0.67	0.03	-0.02	0.08	26.10	1.22	0.24	0.04	0.70	0.03	0.00	0.05	27.00	2.35	0.03	0.09	0.50
bio6	0.01	0.00	0.02	58.06	2.79	0.01	0.07	0.65	0.01	0.00	0.01	26.09	1.18	0.25	0.04	0.70	0.00	-0.02	0.03	27.00	0.30	0.77	0.00	0.50
bio7	-0.01	-0.02	0.00	57.37	-1.24	0.22	0.02	0.66	-0.01	-0.02	0.00	26.08	-1.09	0.29	0.03	0.70	0.02	0.00	0.04	27.00	1.74	0.09	0.05	0.50
bio8	0.05	0.03	0.07	53.68	4.88	0.00	0.23	0.67	0.04	0.00	0.07	26.04	2.05	0.05	0.10	0.70	0.03	0.01	0.05	27.00	2.44	0.02	0.10	0.50
bio9	0.01	0.00	0.03	58.03	2.55	0.01	0.06	0.66	0.01	0.00	0.02	26.09	1.22	0.23	0.04	0.70	0.00	-0.03	0.02	27.00	-0.17	0.86	0.00	0.50
bio10	0.05	0.03	0.06	53.88	4.82	0.00	0.22	0.66	0.03	0.00	0.07	26.09	1.68	0.10	0.07	0.70	0.03	0.01	0.05	27.00	2.44	0.02	0.10	0.50
bio11	0.01	0.00	0.03	58.03	2.55	0.01	0.06	0.66	0.01	0.00	0.02	26.09	1.22	0.23	0.04	0.70	0.00	-0.03	0.02	27.00	-0.17	0.86	0.00	0.50
bio12	0.00	0.00	0.00	57.24	4.15	0.00	0.14	0.65	0.00	0.00	0.00	26.19	0.27	0.79	0.00	0.71	0.00	0.00	0.00	27.00	1.41	0.17	0.04	0.50
bio13	0.00	0.00	0.00	56.21	4.41	0.00	0.16	0.65	0.00	0.00	0.00	26.11	0.15	0.89	0.00	0.71	0.00	0.00	0.01	27.00	2.01	0.05	0.07	0.50
bio14	0.01	0.00	0.02	59.49	2.55	0.01	0.05	0.65	0.00	-0.01	0.01	26.55	0.20	0.85	0.00	0.71	0.07	-0.02	0.16	27.00	1.50	0.15	0.04	0.50
bio15	-1.04	-1.62	-0.46	56.84	-3.49	0.00	0.11	0.66	-0.26	-1.03	0.51	26.38	-0.66	0.51	0.01	0.71	-0.28	-1.29	0.73	27.00	-0.54	0.59	0.01	0.50
bio16	0.00	0.00	0.00	56.02	4.74	0.00	0.18	0.65	0.00	0.00	0.00	26.02	0.35	0.73	0.00	0.71	0.00	0.00	0.00	27.00	1.75	0.09	0.05	0.50
bio17	0.00	0.00	0.01	59.13	2.70	0.01	0.06	0.65	0.00	0.00	0.00	26.44	0.23	0.82	0.00	0.71	0.01	-0.01	0.03	27.00	1.15	0.26	0.02	0.50
bio18	0.00	0.00	0.00	55.96	4.78	0.00	0.18	0.65	0.00	0.00	0.00	25.99	0.39	0.70	0.00	0.71	0.00	0.00	0.00	27.00	1.75	0.09	0.05	0.50

bio19	0.00	0.00	0.01	59.13	2.70	0.01	0.06	0.65	0.00	0.00	0.00	26.44	0.23	0.82	0.00	0.71	0.01	-0.01	0.03	27.00	1.15	0.26	0.02	0.50
T_{gs}	0.07	0.04	0.09	54.30	4.47	0.00	0.19	0.66	0.03	-0.02	0.08	26.24	1.32	0.20	0.04	0.70	0.04	0.01	0.08	27.00	2.35	0.03	0.09	0.50
P_{gs}	0.00	0.00	0.00	57.37	4.22	0.00	0.14	0.65	0.00	0.00	0.00	26.20	0.27	0.79	0.00	0.71	0.00	0.00	0.00	27.00	2.05	0.05	0.07	0.50
AI_{gs}	0.38	0.13	0.64	56.02	2.93	0.00	0.09	0.66	-0.09	-0.48	0.29	26.09	-0.47	0.64	0.01	0.71	-0.14	-0.61	0.33	27.00	-0.58	0.57	0.01	0.50
TN	0.25	-0.23	0.73	56.36	1.02	0.31	0.01	0.66	-0.67	-1.08	-0.27	25.81	-3.22	0.00	0.22	0.70	0.69	-0.48	1.85	27.00	1.15	0.26	0.02	0.50
pH	-0.21	-0.31	-0.11	55.73	-4.21	0.00	0.16	0.65	0.11	-0.14	0.35	26.03	0.86	0.40	0.02	0.71	0.10	-0.19	0.39	27.00	0.64	0.52	0.01	0.50
SAND	-0.02	-0.03	-0.01	55.73	-3.58	0.00	0.13	0.66	-0.01	-0.02	0.01	26.17	-1.02	0.32	0.03	0.71	0.00	-0.01	0.02	27.00	0.11	0.92	0.00	0.50
SILT	0.02	0.00	0.03	54.77	2.33	0.02	0.07	0.66	0.02	0.00	0.04	26.32	1.53	0.14	0.06	0.70	0.00	-0.02	0.01	27.00	-0.42	0.67	0.00	0.50
CLAY	0.03	0.02	0.05	56.59	3.57	0.00	0.11	0.65	0.00	-0.02	0.02	26.14	-0.02	0.99	0.00	0.71	0.01	-0.03	0.05	27.00	0.69	0.49	0.01	0.50
SM	0.30	-1.42	2.02	60.80	0.34	0.73	0.00	0.66	-0.70	-2.11	0.71	27.26	-0.97	0.34	0.02	0.70	1.03	-4.52	6.58	27.00	0.36	0.72	0.00	0.50
BD	-0.01	-0.02	0.00	55.10	-1.54	0.13	0.03	0.66	0.01	0.00	0.03	25.90	1.82	0.08	0.09	0.70	0.01	0.00	0.02	27.00	1.36	0.19	0.03	0.50

Table S11 Relationships between community-weighted skewness of stomatal density (SD_CWS) and environmental variables. All linear regressions are estimated using the linear mixed model with plot nested within sites as a random factor. Slope, the slope of the regression between stomatal trait moments and environments. LL, the lower limit of the 95% confidence interval for slope. UL, the upper limit of the 95% confidence interval for slope. R^2m, marginal R^2 (fixed effects only); R^2c, conditional R^2 (both fixed and random effects). DF, degree of freedom. Different vegetation types and environmental variables were filled with different background colors. See Table S2 for environmental variable abbreviations. Source data are provided as a Source Data file.

	All								Forests								Grasslands							
Y	Slope	LL	UL	DF	T value	P value	R^2c	R^2m	Slope	LL	UL	DF	T value	P value	R^2c	R^2m	Slope	LL	UL	DF	T value	P value	R^2c	R^2m
AI	0.23	-0.41	0.87	60.31	0.71	0.48	0.00	0.36	-0.22	-1.29	0.85	26.21	-0.41	0.69	0.00	0.57	0.63	-1.49	2.75	27.00	0.58	0.57	0.00	0.32
bio1	-0.01	-0.05	0.03	60.91	-0.50	0.62	0.00	0.36	-0.03	-0.08	0.02	26.27	-1.31	0.20	0.04	0.57	0.00	-0.08	0.08	27.00	-0.02	0.98	0.00	0.32
bio2	0.02	-0.10	0.14	62.85	0.35	0.73	0.00	0.36	0.10	-0.05	0.24	26.24	1.30	0.20	0.04	0.57	-0.05	-0.38	0.28	27.00	-0.30	0.76	0.00	0.32
bio3	-0.24	-4.51	4.03	58.02	-0.11	0.91	0.00	0.36	-0.31	-5.80	5.18	26.19	-0.11	0.91	0.00	0.57	0.51	-6.40	7.41	27.00	0.14	0.89	0.00	0.32
bio4	0.03	-0.06	0.11	60.07	0.60	0.55	0.00	0.36	0.06	-0.04	0.16	26.20	1.16	0.26	0.03	0.57	-0.02	-0.17	0.13	27.00	-0.28	0.78	0.00	0.32
bio5	-0.01	-0.06	0.05	51.05	-0.24	0.81	0.00	0.36	-0.08	-0.23	0.06	26.25	-1.14	0.26	0.03	0.57	-0.01	-0.07	0.06	27.00	-0.27	0.79	0.00	0.32
bio6	-0.01	-0.03	0.02	63.99	-0.61	0.54	0.00	0.36	-0.02	-0.04	0.01	26.24	-1.27	0.22	0.04	0.57	0.00	-0.06	0.07	27.00	0.11	0.91	0.00	0.32
bio7	0.01	-0.02	0.03	62.16	0.57	0.57	0.00	0.36	0.02	-0.01	0.05	26.22	1.20	0.24	0.03	0.57	-0.01	-0.07	0.05	27.00	-0.34	0.74	0.00	0.32
bio8	-0.01	-0.06	0.04	52.65	-0.38	0.71	0.00	0.36	-0.09	-0.20	0.02	26.15	-1.66	0.11	0.06	0.57	0.00	-0.07	0.06	27.00	-0.15	0.89	0.00	0.32
bio9	-0.01	-0.04	0.02	63.87	-0.60	0.55	0.00	0.36	-0.02	-0.05	0.01	26.25	-1.27	0.22	0.04	0.57	0.01	-0.07	0.08	27.00	0.20	0.84	0.00	0.32
bio10	-0.01	-0.06	0.04	53.25	-0.27	0.79	0.00	0.36	-0.07	-0.18	0.03	26.26	-1.39	0.18	0.04	0.57	0.00	-0.07	0.06	27.00	-0.15	0.89	0.00	0.32
bio11	-0.01	-0.04	0.02	63.87	-0.60	0.55	0.00	0.36	-0.02	-0.05	0.01	26.25	-1.27	0.22	0.04	0.57	0.01	-0.07	0.08	27.00	0.20	0.84	0.00	0.32
bio12	0.00	0.00	0.00	62.05	0.32	0.75	0.00	0.36	0.00	0.00	0.00	26.34	-0.83	0.41	0.02	0.57	0.00	0.00	0.00	27.00	0.60	0.55	0.01	0.32
bio13	0.00	0.00	0.00	60.10	0.53	0.60	0.00	0.36	0.00	-0.01	0.00	26.22	-0.67	0.51	0.01	0.57	0.00	-0.01	0.01	27.00	0.66	0.52	0.01	0.32
bio14	0.00	-0.02	0.02	68.65	0.19	0.85	0.00	0.36	0.00	-0.03	0.02	26.94	-0.34	0.74	0.00	0.57	0.02	-0.25	0.30	27.00	0.16	0.87	0.00	0.32
bio15	0.56	-0.91	2.03	59.47	0.75	0.46	0.00	0.36	1.36	-0.83	3.56	26.55	1.21	0.24	0.03	0.57	1.52	-1.29	4.32	27.00	1.06	0.30	0.02	0.32
bio16	0.00	0.00	0.00	59.16	0.78	0.44	0.00	0.36	0.00	0.00	0.00	26.10	-0.49	0.63	0.01	0.57	0.00	0.00	0.01	27.00	0.84	0.41	0.01	0.32
bio17	0.00	-0.01	0.01	67.56	0.12	0.91	0.00	0.36	0.00	-0.01	0.00	26.74	-0.51	0.62	0.01	0.57	0.02	-0.05	0.08	27.00	0.48	0.63	0.00	0.32
bio18	0.00	0.00	0.00	58.96	0.77	0.44	0.00	0.36	0.00	0.00	0.00	26.04	-0.51	0.61	0.01	0.57	0.00	0.00	0.01	27.00	0.84	0.41	0.01	0.32

bio19	0.00	-0.01	0.01	67.56	0.12	0.91	0.00	0.36	0.00	-0.01	0.00	26.74	-0.51	0.62	0.01	0.57	0.02	-0.05	0.08	27.00	0.48	0.63	0.00	0.32
T_{gs}	-0.01	-0.09	0.06	54.59	-0.29	0.77	0.00	0.36	-0.07	-0.21	0.07	26.62	-1.03	0.31	0.02	0.57	-0.02	-0.12	0.09	27.00	-0.29	0.78	0.00	0.32
P_{gs}	0.00	0.00	0.00	62.66	0.29	0.77	0.00	0.36	0.00	0.00	0.00	26.36	-0.81	0.42	0.02	0.57	0.00	0.00	0.00	27.00	0.60	0.55	0.01	0.32
AI_{gs}	0.28	-0.35	0.90	58.17	0.86	0.39	0.01	0.36	-0.11	-1.22	1.01	26.20	-0.19	0.85	0.00	0.57	0.38	-0.95	1.72	27.00	0.56	0.58	0.00	0.32
TN	1.14	0.07	2.22	62.17	2.08	0.04	0.03	0.36	1.27	-0.05	2.59	25.94	1.89	0.07	0.08	0.57	0.57	-2.80	3.94	27.00	0.33	0.74	0.00	0.32
pH	-0.16	-0.42	0.09	57.68	-1.25	0.22	0.01	0.36	-0.03	-0.75	0.69	26.08	-0.09	0.93	0.00	0.57	-0.39	-1.20	0.42	27.00	-0.93	0.36	0.01	0.32
SAND	-0.01	-0.04	0.01	56.49	-1.05	0.30	0.01	0.36	-0.01	-0.05	0.04	26.27	-0.38	0.71	0.00	0.57	-0.01	-0.05	0.04	27.00	-0.37	0.71	0.00	0.32
SILT	0.01	-0.03	0.05	53.96	0.56	0.58	0.00	0.36	-0.01	-0.07	0.06	26.46	-0.25	0.80	0.00	0.57	0.01	-0.04	0.06	27.00	0.39	0.70	0.00	0.32
CLAY	0.03	-0.02	0.08	60.44	1.26	0.21	0.01	0.36	0.03	-0.04	0.10	26.34	0.89	0.38	0.02	0.57	0.01	-0.11	0.12	27.00	0.09	0.93	0.00	0.32
SM	-0.38	-4.46	3.71	73.58	-0.18	0.86	0.00	0.36	-1.05	-5.23	3.13	28.10	-0.49	0.63	0.01	0.57	1.37	-14.3	17.08	27.00	0.17	0.87	0.00	0.32
BD	-0.01	-0.04	0.01	56.75	-1.29	0.20	0.01	0.36	-0.02	-0.06	0.01	25.90	-1.22	0.23	0.04	0.57	0.00	-0.04	0.04	27.00	0.02	0.98	0.00	0.32

Table S12 Relationships between community-weighted skewness of stomatal length (SL_CWS) and environmental variables. All linear regressions are estimated using the linear mixed model with plot nested within sites as a random factor. Slope, the slope of the regression between stomatal trait moments and environments. LL, the lower limit of the 95% confidence interval for slope. UL, the upper limit of the 95% confidence interval for slope. R^2m, marginal R^2 (fixed effects only); R^2c, conditional R^2 (both fixed and random effects). DF, degree of freedom. Different vegetation types and environmental variables were filled with different background colors. See Table S2 for environmental variable abbreviations. Source data are provided as a Source Data file.

	All								Forests								Grasslands							
Y	Slope	LL	UL	DF	T value	P value	R^2c	R^2m	Slope	LL	UL	DF	T value	P value	R^2c	R^2m	Slope	LL	UL	DF	T value	P value	R^2c	R^2m
AI	0.80	0.10	1.49	57.41	2.24	0.03	0.04	0.54	1.18	-0.05	2.41	26.26	1.87	0.07	0.08	0.63	-2.61	-4.47	-0.75	27.00	-2.75	0.01	0.11	0.44
bio1	0.09	0.06	0.13	58.53	4.98	0.00	0.17	0.53	0.09	0.04	0.14	26.27	3.37	0.00	0.21	0.63	0.09	0.01	0.16	27.00	2.27	0.03	0.08	0.45
bio2	-0.26	-0.37	-0.14	60.08	-4.38	0.00	0.13	0.53	-0.26	-0.41	-0.11	26.23	-3.35	0.00	0.21	0.63	-0.17	-0.49	0.15	27.00	-1.01	0.32	0.02	0.45
bio3	-1.83	-6.63	2.99	55.20	-0.74	0.46	0.01	0.54	2.11	-4.53	8.76	26.16	0.62	0.54	0.01	0.63	-6.27	-12.7	0.11	27.00	-1.92	0.07	0.06	0.45
bio4	-0.09	-0.19	0.00	57.57	-1.99	0.05	0.04	0.54	-0.16	-0.27	-0.05	26.22	-2.94	0.01	0.17	0.63	0.10	-0.05	0.24	27.00	1.31	0.20	0.03	0.45
bio5	0.12	0.06	0.17	50.62	4.26	0.00	0.17	0.55	0.24	0.08	0.39	26.24	2.97	0.01	0.17	0.63	0.08	0.02	0.14	27.00	2.80	0.01	0.11	0.44
bio6	0.05	0.03	0.07	60.63	4.29	0.00	0.12	0.52	0.05	0.02	0.07	26.25	3.26	0.00	0.20	0.63	0.04	-0.03	0.11	27.00	1.21	0.24	0.02	0.45
bio7	-0.04	-0.07	-0.01	59.04	-2.55	0.01	0.05	0.54	-0.05	-0.08	-0.02	26.23	-3.02	0.01	0.18	0.63	0.03	-0.02	0.09	27.00	1.24	0.23	0.03	0.45
bio8	0.11	0.06	0.16	51.84	4.41	0.00	0.17	0.54	0.18	0.06	0.30	25.94	3.01	0.01	0.18	0.63	0.08	0.02	0.14	27.00	2.55	0.02	0.09	0.44
bio9	0.06	0.03	0.08	60.49	4.10	0.00	0.12	0.53	0.05	0.02	0.09	26.25	3.22	0.00	0.20	0.63	0.04	-0.04	0.11	27.00	0.95	0.35	0.02	0.45
bio10	0.11	0.07	0.16	52.58	4.61	0.00	0.18	0.54	0.19	0.08	0.30	26.26	3.33	0.00	0.21	0.63	0.08	0.02	0.14	27.00	2.55	0.02	0.09	0.44
bio11	0.06	0.03	0.08	60.49	4.10	0.00	0.12	0.53	0.05	0.02	0.09	26.25	3.22	0.00	0.20	0.63	0.04	-0.04	0.11	27.00	0.95	0.35	0.02	0.45
bio12	0.00	0.00	0.00	58.81	2.86	0.01	0.06	0.53	0.00	0.00	0.00	26.40	2.32	0.03	0.12	0.63	0.00	0.00	0.00	27.00	-1.74	0.09	0.05	0.45
bio13	0.00	0.00	0.01	57.45	2.31	0.02	0.05	0.54	0.01	0.00	0.01	26.27	2.06	0.05	0.10	0.63	-0.01	-0.02	0.00	27.00	-2.20	0.04	0.07	0.45
bio14	0.03	0.00	0.05	61.61	2.41	0.02	0.04	0.53	0.02	-0.01	0.05	26.81	1.40	0.17	0.05	0.63	-0.11	-0.37	0.16	27.00	-0.78	0.44	0.01	0.45
bio15	-2.96	-4.44	-1.48	57.30	-3.91	0.00	0.12	0.53	-3.33	-5.76	-0.88	26.58	-2.66	0.01	0.14	0.63	-1.86	-4.59	0.88	27.00	-1.33	0.20	0.03	0.45
bio16	0.00	0.00	0.00	56.86	2.20	0.03	0.04	0.54	0.00	0.00	0.01	26.14	2.08	0.05	0.10	0.63	0.00	-0.01	0.00	27.00	-2.40	0.02	0.09	0.44
bio17	0.01	0.00	0.01	61.23	2.63	0.01	0.05	0.53	0.01	0.00	0.01	26.68	1.61	0.12	0.06	0.63	-0.02	-0.08	0.04	27.00	-0.81	0.43	0.01	0.45

bio18	0.00	0.00	0.00	56.71	2.18	0.03	0.04	0.54	0.00	0.00	0.01	26.07	2.06	0.05	0.10	0.63	0.00	-0.01	0.00	27.00	-2.40	0.02	0.09	0.44
bio19	0.01	0.00	0.01	61.23	2.63	0.01	0.05	0.53	0.01	0.00	0.01	26.68	1.61	0.12	0.06	0.63	-0.02	-0.08	0.04	27.00	-0.81	0.43	0.01	0.45
T_{gs}	0.16	0.09	0.24	53.04	4.19	0.00	0.15	0.54	0.18	0.02	0.34	26.52	2.18	0.04	0.10	0.63	0.13	0.04	0.22	27.00	2.71	0.01	0.10	0.44
P_{gs}	0.00	0.00	0.00	59.20	3.10	0.00	0.07	0.53	0.00	0.00	0.00	26.42	2.36	0.03	0.12	0.63	0.00	0.00	0.00	27.00	-1.26	0.22	0.03	0.45
AI_{gs}	0.45	-0.26	1.15	55.96	1.23	0.22	0.01	0.54	1.04	-0.26	2.34	26.23	1.56	0.13	0.06	0.63	-1.98	-3.07	-0.88	27.00	-3.53	0.00	0.15	0.44
TN	-1.33	-2.54	-0.12	57.31	-2.15	0.04	0.04	0.54	-2.66	-4.04	-1.29	25.95	-3.79	0.00	0.25	0.63	-4.02	-6.99	-1.06	27.00	-2.66	0.01	0.10	0.44
pH	-0.28	-0.57	0.01	55.47	-1.90	0.06	0.03	0.54	-0.58	-1.43	0.27	26.09	-1.34	0.19	0.04	0.63	0.92	0.19	1.66	27.00	2.46	0.02	0.09	0.44
SAND	-0.04	-0.07	-0.02	54.28	-3.41	0.00	0.10	0.54	-0.04	-0.09	0.01	26.16	-1.56	0.13	0.06	0.63	-0.04	-0.08	0.00	27.00	-1.80	0.08	0.05	0.45
SILT	0.04	0.00	0.08	52.74	2.19	0.03	0.05	0.54	0.02	-0.06	0.10	26.35	0.41	0.68	0.00	0.63	0.04	-0.01	0.09	27.00	1.59	0.12	0.04	0.45
CLAY	0.08	0.04	0.13	57.18	3.44	0.00	0.09	0.53	0.09	0.01	0.17	26.26	2.08	0.05	0.10	0.63	0.06	-0.05	0.17	27.00	1.01	0.32	0.02	0.45
SM	-0.74	-5.25	3.77	63.32	-0.32	0.75	0.00	0.54	-0.77	-5.86	4.32	27.66	-0.30	0.77	0.00	0.63	-17.6	-31.6	-3.59	27.00	-2.46	0.02	0.09	0.44
BD	0.02	0.00	0.05	54.35	1.65	0.10	0.03	0.54	0.07	0.03	0.11	25.92	3.53	0.00	0.23	0.63	0.06	0.02	0.09	27.00	2.94	0.01	0.12	0.44

Table S13 Relationships between community-weighted skewness of stomatal pore index (SPI_CWS) and environmental variables. All linear regressions are estimated using the linear mixed model with plot nested within sites as a random factor. Slope, the slope of the regression between stomatal trait moments and environments. LL, the lower limit of the 95% confidence interval for slope. UL, the upper limit of the 95% confidence interval for slope. R^2m, marginal R^2 (fixed effects only); R^2c, conditional R^2 (both fixed and random effects). DF, degree of freedom. Different vegetation types and environmental variables were filled with different background colors. See Table S2 for environmental variable abbreviations. Source data are provided as a Source Data file.

	All								Forests								Grasslands							
Y	Slope	LL	UL	DF	T value	P value	R^2c	R^2m	Slope	LL	UL	DF	T value	P value	R^2c	R^2m	Slope	LL	UL	DF	T value	P value	R^2c	R^2m
AI	-0.01	-0.68	0.67	59.07	-0.02	0.98	0.00	0.52	-0.27	-1.27	0.73	26.19	-0.53	0.60	0.01	0.62	-1.14	-3.59	1.31	27.00	-0.91	0.37	0.02	0.53
bio1	-0.01	-0.05	0.03	59.50	-0.53	0.60	0.00	0.52	-0.01	-0.06	0.04	26.26	-0.33	0.75	0.00	0.62	-0.06	-0.16	0.03	27.00	-1.26	0.22	0.03	0.53
bio2	0.06	-0.06	0.19	60.67	1.01	0.32	0.01	0.52	0.07	-0.06	0.21	26.27	1.04	0.31	0.03	0.62	0.33	-0.04	0.69	27.00	1.75	0.09	0.06	0.53
bio3	5.11	0.81	9.40	57.68	2.33	0.02	0.05	0.51	2.68	-2.36	7.71	26.12	1.04	0.31	0.03	0.62	9.57	2.36	16.78	27.00	2.60	0.02	0.11	0.53
bio4	-0.07	-0.15	0.02	58.69	-1.51	0.14	0.02	0.52	0.00	-0.10	0.09	26.18	-0.02	0.98	0.00	0.62	-0.19	-0.35	-0.04	27.00	-2.39	0.02	0.10	0.53
bio5	-0.05	-0.11	0.00	53.14	-1.81	0.08	0.04	0.52	-0.04	-0.18	0.10	26.24	-0.56	0.58	0.01	0.62	-0.08	-0.15	-0.01	27.00	-2.13	0.04	0.08	0.53
bio6	0.00	-0.02	0.03	61.17	0.16	0.87	0.00	0.52	0.00	-0.03	0.02	26.23	-0.21	0.83	0.00	0.62	0.00	-0.08	0.08	27.00	0.11	0.91	0.00	0.53
bio7	-0.02	-0.04	0.01	60.01	-1.06	0.30	0.01	0.52	0.00	-0.03	0.03	26.20	0.13	0.90	0.00	0.62	-0.06	-0.13	0.00	27.00	-1.98	0.06	0.07	0.53
bio8	-0.05	-0.10	0.00	54.24	-1.82	0.07	0.04	0.52	-0.05	-0.15	0.06	26.16	-0.88	0.39	0.02	0.62	-0.08	-0.15	-0.01	27.00	-2.18	0.04	0.08	0.53
bio9	0.00	-0.02	0.03	61.10	0.32	0.75	0.00	0.52	0.00	-0.03	0.03	26.23	-0.18	0.86	0.00	0.62	0.02	-0.07	0.11	27.00	0.47	0.64	0.00	0.53
bio10	-0.05	-0.10	0.01	54.65	-1.72	0.09	0.03	0.52	-0.04	-0.14	0.06	26.27	-0.75	0.46	0.01	0.62	-0.08	-0.15	-0.01	27.00	-2.18	0.04	0.08	0.53
bio11	0.00	-0.02	0.03	61.10	0.32	0.75	0.00	0.52	0.00	-0.03	0.03	26.23	-0.18	0.86	0.00	0.62	0.02	-0.07	0.11	27.00	0.47	0.64	0.00	0.53
bio12	0.00	0.00	0.00	60.14	-0.19	0.85	0.00	0.52	0.00	0.00	0.00	26.32	-0.48	0.64	0.01	0.62	0.00	0.00	0.00	27.00	-1.45	0.16	0.04	0.53
bio13	0.00	0.00	0.00	59.01	0.00	1.00	0.00	0.52	0.00	0.00	0.01	26.20	0.06	0.96	0.00	0.62	-0.01	-0.02	0.00	27.00	-1.41	0.17	0.04	0.53
bio14	0.00	-0.02	0.02	63.91	0.03	0.97	0.00	0.52	0.00	-0.02	0.02	26.81	-0.29	0.77	0.00	0.62	-0.18	-0.49	0.13	27.00	-1.15	0.26	0.03	0.53
bio15	0.82	-0.71	2.36	58.82	1.05	0.30	0.01	0.52	1.06	-1.01	3.13	26.56	1.00	0.33	0.02	0.62	2.66	-0.53	5.85	27.00	1.63	0.11	0.05	0.53
bio16	0.00	0.00	0.00	58.43	-0.13	0.90	0.00	0.52	0.00	0.00	0.00	26.08	-0.12	0.91	0.00	0.62	0.00	-0.01	0.00	27.00	-1.51	0.14	0.04	0.53
bio17	0.00	-0.01	0.01	63.28	0.07	0.94	0.00	0.52	0.00	-0.01	0.01	26.65	-0.23	0.82	0.00	0.62	-0.05	-0.12	0.02	27.00	-1.33	0.20	0.03	0.53

bio18	0.00	0.00	0.00	58.30	-0.14	0.89	0.00	0.52	0.00	0.00	0.00	26.04	-0.12	0.90	0.00	0.62	0.00	-0.01	0.00	27.00	-1.51	0.14	0.04	0.53
bio19	0.00	-0.01	0.01	63.28	0.07	0.94	0.00	0.52	0.00	-0.01	0.01	26.65	-0.23	0.82	0.00	0.62	-0.05	-0.12	0.02	27.00	-1.33	0.20	0.03	0.53
T_{gs}	-0.07	-0.15	0.01	55.63	-1.80	0.08	0.03	0.52	-0.07	-0.20	0.06	26.56	-1.00	0.33	0.02	0.62	-0.13	-0.24	-0.01	27.00	-2.17	0.04	0.08	0.53
P_{gs}	0.00	0.00	0.00	60.48	-0.13	0.90	0.00	0.52	0.00	0.00	0.00	26.34	-0.37	0.72	0.00	0.62	0.00	-0.01	0.00	27.00	-1.66	0.11	0.05	0.53
AI_{gs}	0.06	-0.61	0.72	57.87	0.18	0.86	0.00	0.52	-0.39	-1.43	0.64	26.16	-0.74	0.46	0.01	0.62	0.06	-1.50	1.63	27.00	0.08	0.94	0.00	0.53
TN	0.12	-1.05	1.29	59.45	0.19	0.85	0.00	0.52	0.60	-0.70	1.89	25.99	0.90	0.38	0.02	0.62	-4.45	-8.02	-0.88	27.00	-2.44	0.02	0.10	0.53
pH	-0.07	-0.34	0.20	57.42	-0.51	0.61	0.00	0.52	0.01	-0.67	0.68	26.06	0.02	0.99	0.00	0.62	0.19	-0.77	1.15	27.00	0.39	0.70	0.00	0.53
SAND	0.00	-0.03	0.02	56.86	-0.32	0.75	0.00	0.52	0.00	-0.05	0.04	26.21	-0.23	0.82	0.00	0.62	0.01	-0.04	0.06	27.00	0.39	0.70	0.00	0.53
SILT	0.01	-0.03	0.05	55.45	0.39	0.69	0.00	0.52	-0.02	-0.08	0.04	26.38	-0.55	0.59	0.01	0.62	0.01	-0.04	0.07	27.00	0.46	0.65	0.00	0.53
CLAY	0.00	-0.05	0.05	59.00	0.10	0.92	0.00	0.52	0.03	-0.03	0.10	26.23	0.98	0.33	0.02	0.62	-0.14	-0.26	-0.02	27.00	-2.25	0.03	0.09	0.53
SM	-1.18	-5.38	3.02	67.00	-0.55	0.58	0.00	0.52	-1.75	-5.62	2.11	27.84	-0.89	0.38	0.02	0.62	0.63	-17.7	18.96	27.00	0.07	0.95	0.00	0.53
BD	-0.01	-0.04	0.01	56.66	-0.98	0.33	0.01	0.52	0.00	-0.03	0.04	25.93	0.17	0.87	0.00	0.62	-0.03	-0.07	0.02	27.00	-1.01	0.32	0.02	0.53

Table S14 Relationships between community-weighted kurtosis of stomatal density (SD_CWK) and environmental variables. All linear regressions are estimated using the linear mixed model with plot nested within sites as a random factor. Slope, the slope of the regression between stomatal trait moments and environments. LL, the lower limit of the 95% confidence interval for slope. UL, the upper limit of the 95% confidence interval for slope. R^2m, marginal R^2 (fixed effects only); R^2c, conditional R^2 (both fixed and random effects). DF, degree of freedom. Different vegetation types and environmental variables were filled with different background colors. See Table S2 for environmental variable abbreviations. Source data are provided as a Source Data file.

	All								Forests								Grasslands							
Y	Slope	LL	UL	DF	T value	P value	R^2c	R^2m	Slope	LL	UL	DF	T value	P value	R^2c	R^2m	Slope	LL	UL	DF	T value	P value	R^2c	R^2m
AI	-1.93	-5.55	1.69	68.79	-1.04	0.30	0.01	0.25	-0.76	-5.21	3.70	26.43	-0.33	0.74	0.00	0.43	-1.41	-14.9	12.14	27.00	-0.20	0.84	0.00	0.27
bio1	-0.13	-0.35	0.09	69.62	-1.16	0.25	0.01	0.25	-0.18	-0.39	0.02	26.54	-1.79	0.08	0.06	0.43	0.11	-0.42	0.64	27.00	0.39	0.70	0.00	0.27
bio2	0.35	-0.32	1.02	72.82	1.03	0.30	0.01	0.25	0.41	-0.18	1.01	26.51	1.37	0.18	0.04	0.43	-0.61	-2.70	1.49	27.00	-0.57	0.58	0.00	0.27
bio3	-2.01	-26.2	22.15	64.22	-0.16	0.87	0.00	0.25	-9.83	-32.29	12.63	26.39	-0.86	0.40	0.01	0.43	4.38	-39.5	48.28	27.00	0.19	0.85	0.00	0.27
bio4	0.16	-0.31	0.64	67.55	0.66	0.51	0.00	0.25	0.37	-0.03	0.76	26.40	1.80	0.08	0.06	0.43	-0.32	-1.27	0.63	27.00	-0.66	0.52	0.01	0.27
bio5	-0.12	-0.42	0.18	54.14	-0.79	0.43	0.00	0.25	-0.40	-0.99	0.20	26.51	-1.31	0.20	0.03	0.43	-0.01	-0.43	0.40	27.00	-0.06	0.95	0.00	0.27
bio6	-0.08	-0.21	0.06	74.76	-1.11	0.27	0.01	0.25	-0.10	-0.20	0.01	26.48	-1.78	0.09	0.06	0.43	0.17	-0.26	0.60	27.00	0.75	0.46	0.01	0.27
bio7	0.07	-0.09	0.22	71.22	0.85	0.40	0.00	0.25	0.11	-0.01	0.23	26.43	1.75	0.09	0.06	0.43	-0.13	-0.49	0.24	27.00	-0.69	0.50	0.01	0.27
bio8	-0.14	-0.43	0.14	56.60	-0.99	0.33	0.01	0.25	-0.39	-0.83	0.05	26.34	-1.74	0.09	0.06	0.43	0.00	-0.43	0.42	27.00	-0.01	0.99	0.00	0.27
bio9	-0.09	-0.24	0.07	74.49	-1.07	0.29	0.01	0.25	-0.11	-0.24	0.01	26.49	-1.83	0.08	0.06	0.43	0.20	-0.28	0.67	27.00	0.81	0.42	0.01	0.27
bio10	-0.14	-0.42	0.14	57.45	-0.97	0.33	0.01	0.25	-0.34	-0.77	0.08	26.54	-1.58	0.13	0.05	0.43	0.00	-0.43	0.42	27.00	-0.01	0.99	0.00	0.27
bio11	-0.09	-0.24	0.07	74.49	-1.07	0.29	0.01	0.25	-0.11	-0.24	0.01	26.49	-1.83	0.08	0.06	0.43	0.20	-0.28	0.67	27.00	0.81	0.42	0.01	0.27
bio12	0.00	-0.01	0.00	71.93	-1.31	0.20	0.01	0.25	0.00	-0.01	0.00	26.64	-0.91	0.37	0.02	0.43	0.00	-0.02	0.01	27.00	-0.32	0.75	0.00	0.27
bio13	-0.01	-0.03	0.01	68.70	-1.37	0.17	0.01	0.25	-0.01	-0.03	0.01	26.44	-0.73	0.47	0.01	0.43	-0.02	-0.08	0.04	27.00	-0.64	0.53	0.01	0.27
bio14	-0.05	-0.17	0.06	82.17	-0.89	0.38	0.00	0.25	-0.02	-0.12	0.07	27.55	-0.51	0.61	0.01	0.43	-0.29	-2.02	1.44	27.00	-0.33	0.74	0.00	0.27
bio15	5.28	-2.99	13.57	67.32	1.25	0.22	0.01	0.25	6.64	-2.41	15.66	27.02	1.44	0.16	0.04	0.43	0.09	-18.1	18.30	27.00	0.01	0.99	0.00	0.27
bio16	-0.01	-0.01	0.00	67.20	-1.25	0.22	0.01	0.25	0.00	-0.01	0.01	26.25	-0.63	0.53	0.01	0.43	-0.01	-0.03	0.02	27.00	-0.42	0.68	0.00	0.27
bio17	-0.02	-0.05	0.01	80.63	-1.01	0.32	0.01	0.25	-0.01	-0.03	0.02	27.25	-0.70	0.49	0.01	0.43	-0.03	-0.42	0.36	27.00	-0.14	0.89	0.00	0.27

bio18	-0.01	-0.01	0.00	66.93	-1.26	0.21	0.01	0.25	0.00	-0.01	0.01	26.16	-0.64	0.53	0.01	0.43	-0.01	-0.03	0.02	27.00	-0.42	0.68	0.00	0.27
bio19	-0.02	-0.05	0.01	80.63	-1.01	0.32	0.01	0.25	-0.01	-0.03	0.02	27.25	-0.70	0.49	0.01	0.43	-0.03	-0.42	0.36	27.00	-0.14	0.89	0.00	0.27
T_{gs}	-0.15	-0.58	0.27	59.19	-0.70	0.49	0.00	0.25	-0.31	-0.89	0.27	27.04	-1.05	0.30	0.02	0.43	0.06	-0.61	0.73	27.00	0.17	0.87	0.00	0.27
P_{gs}	0.00	-0.01	0.00	72.99	-1.36	0.18	0.01	0.25	0.00	-0.01	0.00	26.68	-0.95	0.35	0.02	0.43	0.00	-0.02	0.01	27.00	-0.45	0.66	0.00	0.27
AI_{gs}	-1.42	-4.97	2.14	64.98	-0.78	0.44	0.00	0.25	0.04	-4.59	4.67	26.41	0.02	0.99	0.00	0.43	-0.33	-8.85	8.20	27.00	-0.07	0.94	0.00	0.27
TN	1.02	-5.32	7.36	69.61	0.31	0.75	0.00	0.25	5.33	-0.12	10.78	26.05	1.91	0.07	0.07	0.43	-2.30	-23.8	19.15	27.00	-0.21	0.84	0.00	0.27
pH	0.53	-0.94	2.00	64.69	0.71	0.48	0.00	0.25	0.34	-2.64	3.33	26.21	0.23	0.82	0.00	0.43	-2.09	-7.27	3.10	27.00	-0.79	0.44	0.01	0.27
SAND	0.00	-0.14	0.14	61.99	0.05	0.96	0.00	0.25	0.01	-0.17	0.20	26.47	0.13	0.89	0.00	0.43	-0.14	-0.41	0.14	27.00	-0.97	0.34	0.01	0.27
SILT	0.04	-0.16	0.25	57.99	0.42	0.68	0.00	0.25	-0.05	-0.32	0.22	26.78	-0.40	0.70	0.00	0.43	0.18	-0.13	0.50	27.00	1.14	0.26	0.02	0.27
CLAY	-0.09	-0.35	0.18	67.82	-0.64	0.52	0.00	0.25	0.03	-0.26	0.33	26.54	0.22	0.83	0.00	0.43	-0.02	-0.75	0.71	27.00	-0.05	0.96	0.00	0.27
SM	-8.27	-31.9	15.37	89.26	-0.69	0.50	0.00	0.25	-3.00	-20.54	14.53	29.35	-0.33	0.74	0.00	0.43	-36.1	-135	62.92	27.00	-0.71	0.48	0.01	0.27
BD	0.01	-0.12	0.14	62.07	0.17	0.87	0.00	0.25	-0.13	-0.29	0.03	25.96	-1.60	0.12	0.05	0.43	0.02	-0.25	0.29	27.00	0.15	0.88	0.00	0.27

Table S15 Relationships between community-weighted kurtosis of stomatal length (SL_CWK) and environmental variables. All linear regressions are estimated using the linear mixed model with plot nested within sites as a random factor. Slope, the slope of the regression between stomatal trait moments and environments. LL, the lower limit of the 95% confidence interval for slope. UL, the upper limit of the 95% confidence interval for slope. R^2m, marginal R^2 (fixed effects only); R^2c, conditional R^2 (both fixed and random effects). DF, degree of freedom. Different vegetation types and environmental variables were filled with different background colors. See Table S2 for environmental variable abbreviations. Source data are provided as a Source Data file.

	All								Forests								Grasslands							
Y	Slope	LL	UL	DF	T value	P value	R^2c	R^2m	Slope	LL	UL	DF	T value	P value	R^2c	R^2m	Slope	LL	UL	DF	T value	P value	R^2c	R^2m
AI	2.71	0.20	5.22	58.34	2.11	0.04	0.03	0.31	2.63	-2.37	7.63	26.64	1.03	0.31	0.01	0.18	-2.12	-9.07	4.84	27.00	-0.60	0.56	0.01	0.34
bio1	0.12	-0.04	0.28	58.66	1.48	0.15	0.01	0.31	0.13	-0.11	0.36	26.81	1.02	0.32	0.01	0.18	-0.10	-0.37	0.17	27.00	-0.73	0.47	0.01	0.34
bio2	-0.42	-0.89	0.05	61.32	-1.74	0.09	0.02	0.31	-0.43	-1.11	0.25	26.69	-1.25	0.22	0.02	0.18	0.68	-0.38	1.73	27.00	1.25	0.22	0.02	0.34
bio3	9.74	-7.38	26.80	54.25	1.12	0.27	0.01	0.31	4.27	-21.70	30.25	26.55	0.32	0.75	0.00	0.18	23.17	2.22	44.12	27.00	2.16	0.04	0.06	0.34
bio4	-0.42	-0.74	-0.09	56.45	-2.53	0.01	0.04	0.30	-0.31	-0.77	0.16	26.55	-1.28	0.21	0.02	0.18	-0.50	-0.96	-0.05	27.00	-2.16	0.04	0.06	0.34
bio5	0.00	-0.22	0.22	47.17	0.01	0.99	0.00	0.31	0.29	-0.41	0.98	26.75	0.81	0.43	0.01	0.18	-0.16	-0.37	0.04	27.00	-1.57	0.13	0.03	0.34
bio6	0.10	0.01	0.19	62.32	2.11	0.04	0.03	0.30	0.08	-0.05	0.21	26.68	1.24	0.23	0.02	0.18	0.04	-0.18	0.27	27.00	0.36	0.72	0.00	0.34
bio7	-0.13	-0.23	-0.02	59.38	-2.42	0.02	0.03	0.30	-0.09	-0.23	0.05	26.60	-1.24	0.23	0.02	0.18	-0.16	-0.34	0.02	27.00	-1.73	0.09	0.04	0.34
bio8	0.02	-0.19	0.22	48.93	0.14	0.89	0.00	0.31	0.16	-0.36	0.69	26.35	0.61	0.54	0.01	0.18	-0.17	-0.38	0.04	27.00	-1.62	0.12	0.04	0.34
bio9	0.12	0.01	0.22	61.95	2.14	0.04	0.03	0.30	0.09	-0.06	0.23	26.71	1.16	0.26	0.02	0.18	0.10	-0.15	0.34	27.00	0.77	0.45	0.01	0.34
bio10	0.02	-0.18	0.23	49.65	0.23	0.82	0.00	0.31	0.17	-0.33	0.67	26.84	0.66	0.51	0.01	0.18	-0.17	-0.38	0.04	27.00	-1.62	0.12	0.04	0.34
bio11	0.12	0.01	0.22	61.95	2.14	0.04	0.03	0.30	0.09	-0.06	0.23	26.71	1.16	0.26	0.02	0.18	0.10	-0.15	0.34	27.00	0.77	0.45	0.01	0.34
bio12	0.00	0.00	0.00	60.18	1.64	0.11	0.02	0.31	0.00	0.00	0.01	27.08	0.65	0.52	0.01	0.18	0.00	-0.01	0.00	27.00	-1.26	0.22	0.02	0.34
bio13	0.01	-0.01	0.02	57.77	1.08	0.29	0.01	0.31	0.00	-0.02	0.03	26.71	0.27	0.79	0.00	0.18	-0.03	-0.06	0.00	27.00	-1.81	0.08	0.04	0.34
bio14	0.03	-0.05	0.11	67.06	0.77	0.45	0.00	0.31	-0.01	-0.11	0.10	28.94	-0.10	0.92	0.00	0.18	-0.79	-1.64	0.05	27.00	-1.84	0.08	0.04	0.34
bio15	-5.00	-10.9	0.84	57.17	-1.67	0.10	0.02	0.31	-5.89	-16.42	4.69	27.79	-1.09	0.28	0.02	0.18	2.69	-6.67	12.04	27.00	0.56	0.58	0.00	0.34
bio16	0.00	0.00	0.01	56.78	1.23	0.22	0.01	0.31	0.00	-0.01	0.01	26.33	0.38	0.71	0.00	0.18	-0.01	-0.02	0.00	27.00	-1.61	0.12	0.04	0.34

bio17	0.01	-0.01	0.03	65.87	0.88	0.38	0.00	0.31	0.00	-0.03	0.03	28.33	-0.06	0.96	0.00	0.18	-0.13	-0.32	0.07	27.00	-1.27	0.22	0.02	0.34
bio18	0.00	0.00	0.01	56.57	1.22	0.23	0.01	0.31	0.00	-0.01	0.01	26.16	0.38	0.71	0.00	0.18	-0.01	-0.02	0.00	27.00	-1.61	0.12	0.04	0.34
bio19	0.01	-0.01	0.03	65.87	0.88	0.38	0.00	0.31	0.00	-0.03	0.03	28.33	-0.06	0.96	0.00	0.18	-0.13	-0.32	0.07	27.00	-1.27	0.22	0.02	0.34
T_{gs}	0.04	-0.27	0.35	51.09	0.26	0.80	0.00	0.31	0.12	-0.56	0.80	27.78	0.34	0.74	0.00	0.18	-0.23	-0.57	0.10	27.00	-1.36	0.18	0.03	0.34
P_{gs}	0.00	0.00	0.00	60.99	1.68	0.10	0.02	0.31	0.00	0.00	0.01	27.15	0.75	0.46	0.01	0.18	-0.01	-0.02	0.00	27.00	-1.72	0.10	0.04	0.34
AI_{gs}	2.81	0.36	5.26	55.47	2.24	0.03	0.03	0.31	2.72	-2.48	7.90	26.59	1.02	0.32	0.01	0.18	0.81	-3.58	5.21	27.00	0.36	0.72	0.00	0.34
TN	1.13	-3.38	5.65	58.34	0.49	0.63	0.00	0.31	-1.01	-7.63	5.62	25.98	-0.30	0.77	0.00	0.18	-6.52	-17.3	4.30	27.00	-1.18	0.25	0.02	0.34
pH	-1.39	-2.38	-0.40	55.22	-2.75	0.01	0.05	0.30	-2.91	-6.14	0.31	26.17	-1.77	0.09	0.04	0.18	-0.70	-3.40	1.99	27.00	-0.51	0.61	0.00	0.34
SAND	-0.06	-0.16	0.04	52.72	-1.18	0.24	0.01	0.31	0.06	-0.15	0.28	26.78	0.59	0.56	0.00	0.18	-0.04	-0.19	0.10	27.00	-0.57	0.57	0.00	0.34
SILT	0.05	-0.10	0.20	49.97	0.68	0.50	0.00	0.31	-0.20	-0.50	0.11	27.36	-1.27	0.21	0.02	0.18	0.09	-0.08	0.25	27.00	1.03	0.31	0.02	0.34
CLAY	0.13	-0.06	0.31	57.50	1.35	0.18	0.01	0.31	0.07	-0.27	0.41	26.83	0.42	0.68	0.00	0.18	-0.16	-0.53	0.21	27.00	-0.83	0.42	0.01	0.34
SM	1.04	-15.7	17.76	72.48	0.12	0.90	0.00	0.31	-1.99	-22.64	18.62	32.90	-0.19	0.85	0.00	0.18	-11.0	-62.4	40.49	27.00	-0.42	0.68	0.00	0.34
BD	-0.05	-0.14	0.05	52.65	-0.97	0.34	0.01	0.31	0.10	-0.08	0.29	25.81	1.09	0.29	0.02	0.18	-0.06	-0.20	0.08	27.00	-0.83	0.42	0.01	0.34

Table S16 Relationships between community-weighted kurtosis of stomatal pore index (SPI_CWK) and environmental variables. All linear regressions are estimated using the linear mixed model with plot nested within sites as a random factor. Slope, the slope of the regression between stomatal trait moments and environments. LL, the lower limit of the 95% confidence interval for slope. UL, the upper limit of the 95% confidence interval for slope. R^2m, marginal R^2 (fixed effects only); R^2c, conditional R^2 (both fixed and random effects). DF, degree of freedom. Different vegetation types and environmental variables were filled with different background colors. See Table S2 for environmental variable abbreviations. Source data are provided as a Source Data file.

	All								Forests								Grasslands							
Y	Slope	LL	UL	DF	T value	P value	R^2c	R^2m	Slope	LL	UL	DF	T value	P value	R^2c	R^2m	Slope	LL	UL	DF	T value	P value	R^2c	R^2m
AI	-1.53	-4.17	1.10	68.39	-1.14	0.26	0.01	0.29	-1.49	-4.27	1.29	26.43	-1.05	0.30	0.02	0.33	-0.89	-11.3	9.52	27.00	-0.17	0.87	0.00	0.34
bio1	-0.16	-0.32	0.00	69.54	-1.96	0.05	0.02	0.29	-0.13	-0.25	0.00	26.72	-1.95	0.06	0.06	0.33	-0.22	-0.62	0.18	27.00	-1.10	0.28	0.02	0.34
bio2	0.48	0.00	0.96	72.94	1.96	0.05	0.02	0.29	0.46	0.11	0.81	26.83	2.57	0.02	0.10	0.33	0.61	-1.00	2.21	27.00	0.74	0.47	0.01	0.34
bio3	11.88	-5.55	29.24	64.32	1.34	0.19	0.01	0.29	2.72	-11.72	17.15	26.27	0.37	0.72	0.00	0.33	22.35	-10.3	55.04	27.00	1.34	0.19	0.02	0.34
bio4	-0.01	-0.35	0.34	66.81	-0.04	0.97	0.00	0.29	0.20	-0.06	0.46	26.49	1.52	0.14	0.04	0.33	-0.47	-1.18	0.24	27.00	-1.29	0.21	0.02	0.34
bio5	-0.25	-0.46	-0.04	55.43	-2.32	0.02	0.04	0.29	-0.33	-0.70	0.04	26.61	-1.72	0.10	0.05	0.33	-0.23	-0.54	0.07	27.00	-1.50	0.14	0.03	0.33
bio6	-0.07	-0.17	0.03	73.61	-1.40	0.17	0.01	0.29	-0.06	-0.13	0.00	26.64	-1.81	0.08	0.05	0.33	-0.05	-0.38	0.28	27.00	-0.28	0.78	0.00	0.34
bio7	0.02	-0.09	0.14	70.11	0.43	0.67	0.00	0.29	0.07	-0.01	0.15	26.56	1.70	0.10	0.05	0.33	-0.15	-0.43	0.13	27.00	-1.07	0.29	0.02	0.34
bio8	-0.25	-0.45	-0.05	57.87	-2.48	0.02	0.04	0.29	-0.31	-0.58	-0.04	26.39	-2.25	0.03	0.08	0.33	-0.24	-0.55	0.07	27.00	-1.51	0.14	0.03	0.33
bio9	-0.07	-0.19	0.04	73.28	-1.25	0.21	0.01	0.29	-0.07	-0.15	0.01	26.64	-1.79	0.09	0.05	0.33	-0.01	-0.38	0.36	27.00	-0.03	0.97	0.00	0.34
bio10	-0.25	-0.44	-0.05	58.72	-2.48	0.02	0.04	0.29	-0.30	-0.56	-0.04	26.74	-2.24	0.03	0.08	0.33	-0.24	-0.55	0.07	27.00	-1.51	0.14	0.03	0.33
bio11	-0.07	-0.19	0.04	73.28	-1.25	0.21	0.01	0.29	-0.07	-0.15	0.01	26.64	-1.79	0.09	0.05	0.33	-0.01	-0.38	0.36	27.00	-0.03	0.97	0.00	0.34
bio12	0.00	0.00	0.00	71.17	-1.47	0.15	0.01	0.29	0.00	0.00	0.00	26.79	-1.41	0.17	0.03	0.33	0.00	-0.02	0.01	27.00	-0.71	0.48	0.01	0.34
bio13	-0.01	-0.02	0.00	68.10	-1.38	0.17	0.01	0.29	-0.01	-0.02	0.01	26.40	-0.72	0.48	0.01	0.33	-0.02	-0.07	0.02	27.00	-1.00	0.33	0.01	0.34
bio14	-0.05	-0.13	0.03	80.13	-1.17	0.24	0.01	0.29	-0.03	-0.09	0.02	28.00	-1.14	0.27	0.02	0.33	-0.82	-2.11	0.48	27.00	-1.23	0.23	0.02	0.34
bio15	6.69	0.80	12.58	68.01	2.22	0.03	0.03	0.29	6.97	1.59	12.34	27.59	2.53	0.02	0.09	0.32	7.98	-5.69	21.65	27.00	1.14	0.26	0.02	0.34
bio16	0.00	-0.01	0.00	66.86	-1.33	0.19	0.01	0.29	0.00	-0.01	0.00	26.16	-0.81	0.42	0.01	0.33	-0.01	-0.03	0.01	27.00	-0.76	0.45	0.01	0.34
bio17	-0.01	-0.04	0.01	78.59	-1.13	0.26	0.01	0.29	-0.01	-0.02	0.01	27.59	-1.11	0.28	0.02	0.33	-0.10	-0.40	0.20	27.00	-0.67	0.51	0.01	0.34
bio18	0.00	-0.01	0.00	66.60	-1.33	0.19	0.01	0.29	0.00	-0.01	0.00	26.04	-0.80	0.43	0.01	0.33	-0.01	-0.03	0.01	27.00	-0.76	0.45	0.01	0.34

bio19	-0.01	-0.04	0.01	78.59	-1.13	0.26	0.01	0.29	-0.01	-0.02	0.01	27.59	-1.11	0.28	0.02	0.33	-0.10	-0.40	0.20	27.00	-0.67	0.51	0.01	0.34
T_{gs}	-0.35	-0.64	-0.05	60.38	-2.26	0.03	0.04	0.29	-0.33	-0.68	0.03	27.32	-1.81	0.08	0.05	0.33	-0.36	-0.85	0.14	27.00	-1.39	0.17	0.03	0.34
P_{gs}	0.00	0.00	0.00	72.04	-1.52	0.13	0.01	0.29	0.00	0.00	0.00	26.84	-1.34	0.19	0.03	0.33	-0.01	-0.02	0.01	27.00	-1.06	0.30	0.02	0.34
AI_{gs}	-0.85	-3.46	1.75	65.05	-0.64	0.52	0.00	0.29	-1.41	-4.30	1.49	26.38	-0.95	0.35	0.02	0.33	1.69	-4.84	8.21	27.00	0.51	0.62	0.00	0.34
TN	1.40	-3.21	6.02	69.01	0.60	0.55	0.00	0.29	4.37	1.07	7.67	25.93	2.59	0.02	0.10	0.33	-0.48	-17.0	16.01	27.00	-0.06	0.95	0.00	0.34
pH	0.39	-0.68	1.47	64.55	0.72	0.48	0.00	0.29	0.79	-1.09	2.67	26.10	0.82	0.42	0.01	0.33	-1.39	-5.39	2.60	27.00	-0.68	0.50	0.01	0.34
SAND	0.04	-0.07	0.14	62.56	0.67	0.50	0.00	0.29	0.05	-0.07	0.16	26.43	0.76	0.46	0.01	0.33	-0.02	-0.24	0.19	27.00	-0.20	0.85	0.00	0.34
SILT	-0.03	-0.18	0.12	59.18	-0.43	0.67	0.00	0.29	-0.08	-0.25	0.09	26.76	-0.94	0.36	0.02	0.33	0.03	-0.21	0.28	27.00	0.26	0.80	0.00	0.34
CLAY	-0.07	-0.26	0.12	67.50	-0.71	0.48	0.00	0.29	-0.02	-0.21	0.17	26.51	-0.18	0.85	0.00	0.33	-0.02	-0.58	0.54	27.00	-0.07	0.94	0.00	0.34
SM	-7.14	-24.2	9.93	86.23	-0.82	0.42	0.00	0.29	-4.41	-15.57	6.74	30.49	-0.77	0.45	0.01	0.33	-20.4	-96.7	56.02	27.00	-0.52	0.61	0.00	0.34
BD	-0.01	-0.11	0.08	62.33	-0.29	0.77	0.00	0.29	-0.05	-0.15	0.06	25.80	-0.87	0.39	0.01	0.33	-0.09	-0.29	0.12	27.00	-0.83	0.42	0.01	0.34

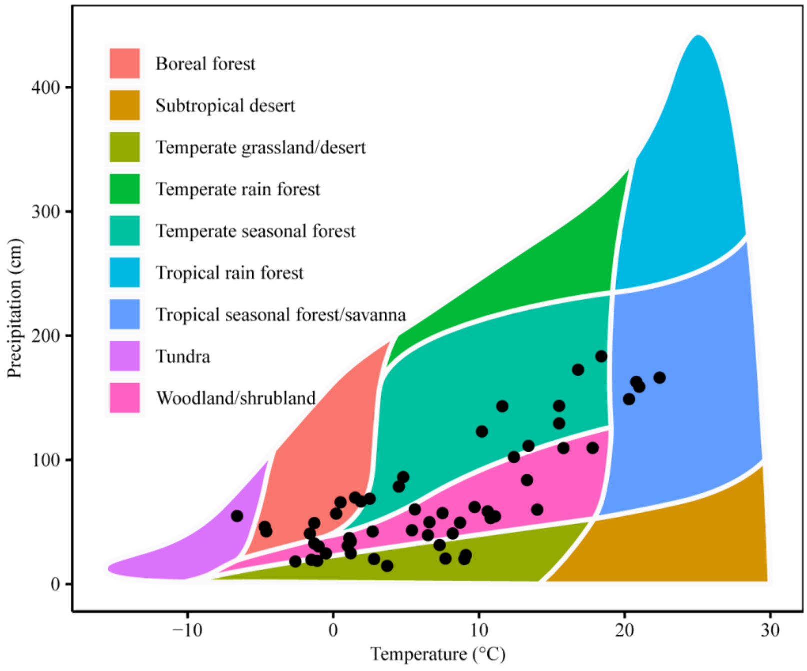

Figure S1 Environmental locations across Whittaker's biomes

Black points represent our sampling sites. Source data are provided as a Source Data file.

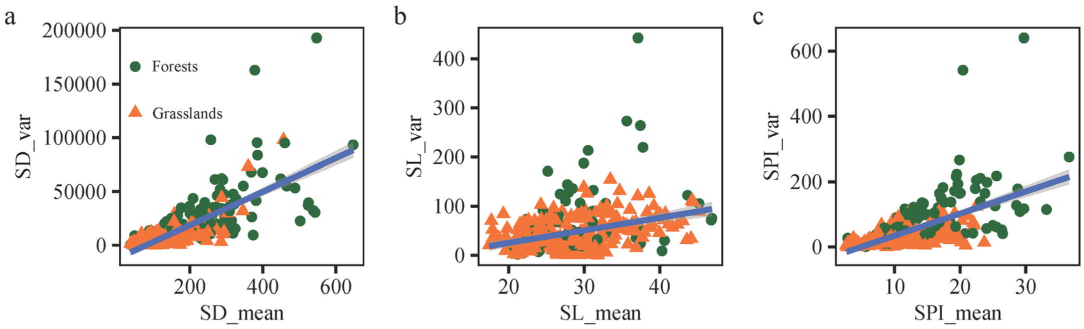

Figure S2 Relationships between community-weighted mean and variance of stomatal traits. The circle and triangle represent forest and grassland sampling sites, respectively. The solid lines represent the linear regressions, while the gray shading indicates the 95% confidence interval. All linear regressions are estimated ordinary least squares method. **a** Relationships between community-weighted mean and variance of stomatal density (two-sided Pearson r-squared = 0.553, $p < 0.001$). **b** Relationships between community-weighted mean and variance of stomatal length (two-sided Pearson r-squared = 0.112, $p < 0.001$). **c** Relationships between community-weighted mean and variance of stomatal pore index (two-sided Pearson r-squared = 0.389, $p < 0.001$). SD_mean, community-weighted mean of stomatal density; SL_mean, community-weighted mean of stomatal length; SPI_mean, community-weighted mean of stomatal pore index. SD_var, community-weighted variance of stomatal density; SL_var, community-weighted variance of stomatal length; SPI_var, community-weighted variance of stomatal pore index. Source data are provided as a Source Data file.

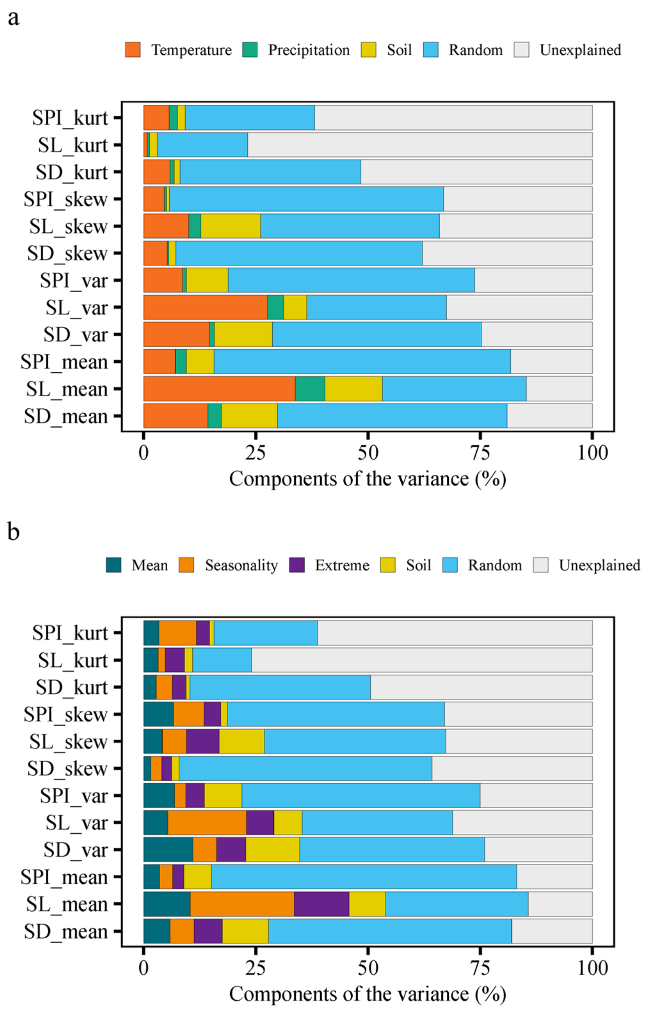

Figure S3 Variance partition of stomatal trait moments in forests.

Different colors represent different environmental groups, random factor, and unexplained variation. Statistical analysis was performed using linear mixed effects models with environmental groups as fixed factors, and plot nested within sites as random factors. **a** The relative importance of temperature factors, precipitation factors, soil factors, and random factors for stomatal trait moments (two-tailed statistical test). **b** The relative importance of climatic mean, climatic seasonality, climatic extreme, soil factors, and random factors for stomatal trait moments (two-tailed statistical test). SD_mean, community-weighted mean of stomatal density; SL_mean, community-weighted mean of stomatal length; SPI_mean, community-weighted mean of stomatal pore index. SD_var, community-weighted variance of stomatal density; SL_var, community-weighted variance of stomatal length; SPI_var, community-weighted variance of stomatal pore index. SD_skew, community-weighted skewness of stomatal density; SL_skew, community-weighted skewness of stomatal length; SPI_ skew, community-weighted skewness of stomatal pore index. SD_kurt, community-weighted kurtosis of stomatal density; SL_kurt, community-weighted kurtosis of stomatal length; SPI_kurt, community-weighted kurtosis of stomatal pore index. Source data are provided as a Source Data file.

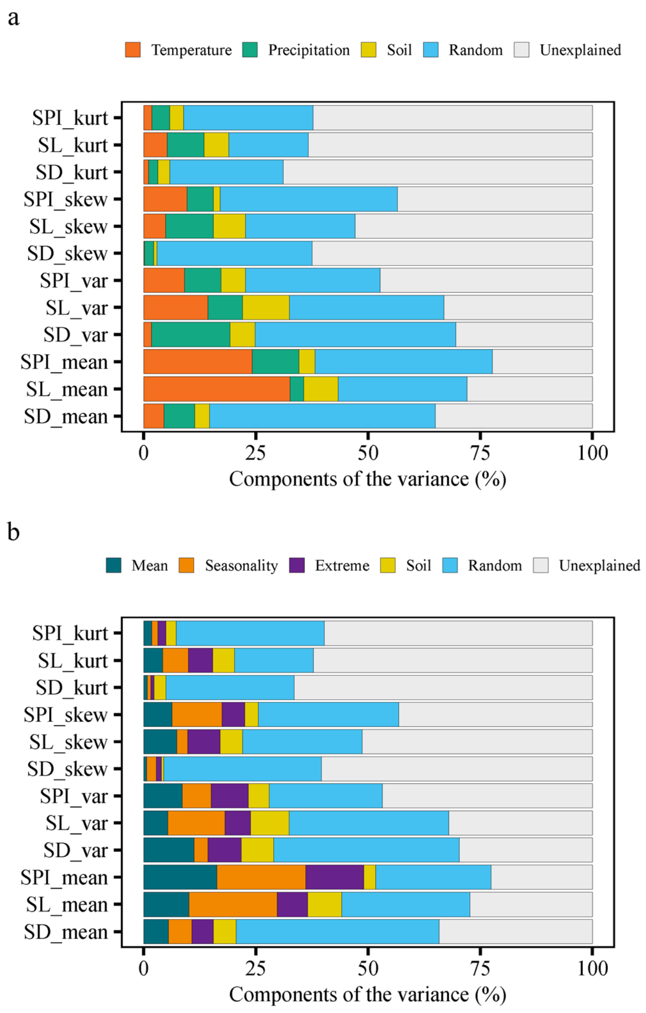

Figure S4 Variance partition of stomatal trait moments in grasslands.

Different colors represent different environmental groups, random factor, and unexplained variation. Statistical analysis was performed using linear mixed effects models with environmental groups as fixed factors, and plot nested within sites as random factors. **a** The relative importance of temperature factors, precipitation factors, soil factors, and random factors for stomatal trait moments (two-tailed statistical test). **b** The relative importance of climatic mean, climatic seasonality, climatic extreme, soil factors, and random factors for stomatal trait moments (two-tailed statistical test). SD_mean, community-weighted mean of stomatal density; SL_mean, community-weighted mean of stomatal length; SPI_mean, community-weighted mean of stomatal pore index. SD_var, community-weighted variance of stomatal density; SL_var, community-weighted variance of stomatal length; SPI_var, community-weighted variance of stomatal pore index. SD_skew, community-weighted skewness of stomatal density; SL_skew, community-weighted skewness of stomatal length; SPI_ skew, community-weighted skewness of stomatal pore index. SD_kurt, community-weighted kurtosis of stomatal density; SL_kurt, community-weighted kurtosis of stomatal length; SPI_kurt, community-weighted kurtosis of stomatal pore index. Source data are provided as a Source Data file.
