# Supplementary material for: Relationships of stomatal morphology to the environment across plant communities
Source: Nat Commun. 2023 Oct 19;14:6629. doi: 10.1038/s41467-023-42136-2 (PMC10587080; doi:10.1038/s41467-023-42136-2)
Supplement: Supplementary file 2 — Reporting Summary [file 41467_2023_42136_MOESM2_ESM.pdf]

nature portfolio

Corresponding author(s): Nianpeng He

Last updated by author(s): Aug 23, 2023

Reporting Summary

Nature Portfolio wishes to improve the reproducibility of the work that we publish. This form provides structure for consistency and transparency in reporting. For further information on Nature Portfolio policies, see our Editorial Policies and the Editorial Policy Checklist.

Statistics

For all statistical analyses, confirm that the following items are present in the figure legend, table legend, main text, or Methods section.

n/a	Confirmed	
☐	☒	The exact sample size (*n*) for each experimental group/condition, given as a discrete number and unit of measurement
☐	☒	A statement on whether measurements were taken from distinct samples or whether the same sample was measured repeatedly
☐	☒	The statistical test(s) used AND whether they are one- or two-sided<br>*Only common tests should be described solely by name; describe more complex techniques in the Methods section.*
☒	☐	A description of all covariates tested
☐	☒	A description of any assumptions or corrections, such as tests of normality and adjustment for multiple comparisons
☐	☒	A full description of the statistical parameters including central tendency (e.g. means) or other basic estimates (e.g. regression coefficient) AND variation (e.g. standard deviation) or associated estimates of uncertainty (e.g. confidence intervals)
☐	☒	For null hypothesis testing, the test statistic (e.g. *F*, *t*, *r*) with confidence intervals, effect sizes, degrees of freedom and *P* value noted<br>*Give P values as exact values whenever suitable.*
☒	☐	For Bayesian analysis, information on the choice of priors and Markov chain Monte Carlo settings
☒	☐	For hierarchical and complex designs, identification of the appropriate level for tests and full reporting of outcomes
☒	☐	Estimates of effect sizes (e.g. Cohen's *d*, Pearson's *r*), indicating how they were calculated

Our web collection on statistics for biologists contains articles on many of the points above.

Software and code

Policy information about availability of computer code

Data collection	Stomatal traits were measured using a scanning electron microscope (S-3400N, Hitachi, Japan).
Data analysis	All analyses were conducted in R version 4.1.1 (https://www.r-project.org/). We also used following open-source R packages for statistical analyses: lmer4, MuMIn, glmm.hp.

For manuscripts utilizing custom algorithms or software that are central to the research but not yet described in published literature, software must be made available to editors and reviewers. We strongly encourage code deposition in a community repository (e.g. GitHub). See the Nature Portfolio guidelines for submitting code & software for further information.

Data

Policy information about availability of data

All manuscripts must include a data availability statement. This statement should provide the following information, where applicable:

- Accession codes, unique identifiers, or web links for publicly available datasets
- A description of any restrictions on data availability
- For clinical datasets or third party data, please ensure that the statement adheres to our policy

The raw and processed stomatal trait data at the community level generated in this study have been deposited in the figshare database and can be accessed at https://doi.org/10.6084/m9.figshare.24015252.

Research involving human participants, their data, or biological material

Policy information about studies with human participants or human data. See also policy information about sex, gender (identity/presentation), and sexual orientation and race, ethnicity and racism.

Reporting on sex and gender	N/A
Reporting on race, ethnicity, or other socially relevant groupings	N/A
Population characteristics	N/A
Recruitment	N/A
Ethics oversight	N/A

Note that full information on the approval of the study protocol must also be provided in the manuscript.

Field-specific reporting

Please select the one below that is the best fit for your research. If you are not sure, read the appropriate sections before making your selection.

☐ Life sciences ☐ Behavioural & social sciences ☒ Ecological, evolutionary & environmental sciences

For a reference copy of the document with all sections, see nature.com/documents/nr-reporting-summary-flat.pdf

Ecological, evolutionary & environmental sciences study design

All studies must disclose on these points even when the disclosure is negative.

Study description	We aimed to test stomatal trait- relationship at the community level
Research sample	We measured stomatal morphology of 4492 species-site combinations in 340 vegetation plots spanning latitude 18.7 °N - 53.3 °N and longitude 81.2 °E - 128.9 °E and calculated their community-weighted values for mean, variance, skewness, and kurtosis.
Sampling strategy	Sampling plots were located within well-protected national nature reserves or national ecological observatory sites, in areas of relatively continuous vegetation representative of the given site.
Data collection	20 – 40 mature leaves were collected from at least four healthy individuals and mixed as a composite sample.<br>For each species, three small pieces were selected from the pooled sample. Each replicate was photographed twice at different positions on the lower surface (forest species) or both surfaces (grassland species); thus, we imaged each forest species six times and each grassland species six or 12 times.
Timing and spatial scale	The field survey was conducted in July and August from 2013 to 2018, the peak period of vegetation growth.<br>340 vegetation plots spanning latitude 18.7 °N - 53.3 °N and longitude 81.2 °E - 128.9 °E
Data exclusions	No data were excluded
Reproducibility	In forests, three or four experimental plots (30 m × 40 m) were established.<br>In grasslands, eight plots (1 m × 1 m) in each site were established.<br>R codes and stomatal trait moments have been deposited in the figshare database and can be accessed at https://doi.org/10.6084/m9.figshare.24015252.
Randomization	We randomized stomatal trait values across all species 500 times. For each run we calculated stomatal trait moments with the randomized trait values, but retaining the species set and their abundances in each plant community intact. For each stomatal trait, we calculated standardized effect sizes (SESs) for the variance in CWMs and for the mean in CWVs, CWSs, and CWKs.
Blinding	Blinding was not applicable since this is a field investigation experiment.

Did the study involve field work? ☒ Yes ☐ No

Field work, collection and transport

Field conditions	The mean annual temperature ranges from –6.6 to 22.4 °C, and mean annual precipitation (MAP) ranges from 146 to 1834 mm
Location	The study sites extend from 18.7 to 51.8 °N in latitude, and from 81.2 to 128.9 °E in longitude, and represent most of the vegetation

	types in the northern hemisphere, including cold-temperate coniferous forest, temperate coniferous and broad-leaved mixed forest, warm temperate deciduous broad-leaved forest, subtropical evergreen broad-leaved forest, tropical rain forest, meadow steppe, typical steppe, and desert steppe
Access & import/export	Sampling plots were located within well-protected national nature reserves or national ecological observatory sites. All of them are the member of Chinese Ecosystem Research Network (CERN), and we have got permission from CERN.
Disturbance	Sampling plots were located within well-protected national nature reserves or national ecological observatory sites, in areas of relatively continuous vegetation representative of the given site. That is, we minimize human disturbanceference as much as possible

Reporting for specific materials, systems and methods

We require information from authors about some types of materials, experimental systems and methods used in many studies. Here, indicate whether each material, system or method listed is relevant to your study. If you are not sure if a list item applies to your research, read the appropriate section before selecting a response.

Materials & experimental systems

n/a	Involved in the study	
☒	☐	Antibodies
☒	☐	Eukaryotic cell lines
☒	☐	Palaeontology and archaeology
☒	☐	Animals and other organisms
☒	☐	Clinical data
☒	☐	Dual use research of concern
☒	☐	Plants

Methods

n/a	Involved in the study	
☒	☐	ChIP-seq
☒	☐	Flow cytometry
☒	☐	MRI-based neuroimaging
