# Supplementary material for: Relationships of stomatal morphology to the environment across plant communities
Source: Nat Commun. 2023 Oct 19;14:6629. doi: 10.1038/s41467-023-42136-2 (PMC10587080; doi:10.1038/s41467-023-42136-2)
Supplement: Supplementary file 4 — Description of Additional Supplementary Files [file 41467_2023_42136_MOESM4_ESM.pdf]

Description of Additional Supplementary Files

File Name: Supplementary Data 1

Description:

Dataset of Relationships of Stomatal Morphology to Environment across Plant Communities, including stomatal trait moments (mean, variance, skewness, and kurtosis), climate, and soil variables.

File Name: Supplementary Code 1

Description:

R code for Relationships of Stomatal Morphology to Environment across Plant Communities.
